# Quantitative measurement of cell-surface displayed proteins based on split-GFP assembly

**DOI:** 10.1186/s12934-024-02386-1

**Published:** 2024-04-12

**Authors:** Li Zhang, Ling Tan, Meizi Liu, Yunhong Chen, Yu Yang, Yanfei Zhang, Guoping Zhao

**Affiliations:** 1 Tianjin Institute of Industrial Biotechnology, Chinese Academy of Sciences, Tianjin, 300308 China; 2 School of Minerals Processing and Bioengineering, Central South University, Changsha, Hunan 410083 PR China; 3 Haihe Laboratory of Synthetic Biology, Tianjin, 300308 China; 4 National Center of Technology Innovation for Synthetic Biology, Tianjin, 300308 China; 5 CAS-Key Laboratory of Synthetic Biology, CAS Center for Excellence in Molecular Plant Sciences, Institute of Plant Physiology and Ecology, Chinese Academy of Sciences, Shanghai, 200032 China

**Keywords:** Microbial surface display, Split-GFP, Anchor protein, Display efficiency, Quantification of displayed proteins

## Abstract

**Background:**

Microbial cell surface display technology allows immobilizing proteins on the cell surface by fusing them to anchoring motifs, thereby endowing the cells with diverse functionalities. However, the assessment of successful protein display and the quantification of displayed proteins remain challenging. The green fluorescent protein (GFP) can be split into two non-fluorescent fragments, while they spontaneously assemble and emit fluorescence when brought together through complementation. Based on split-GFP assembly, we aim to: (1) confirm the success display of passenger proteins, (2) quantify the number of passenger proteins displayed on individual cells.

**Results:**

In this study, we propose two innovative methods based on split-green fluorescent protein (split-GFP), named GFP1-10/GFP11 and GFP1-9/GFP10-11 assembly, for the purpose of confirming successful display and quantifying the number of proteins displayed on individual cells. We evaluated the display efficiency of SUMO and ubiquitin using different anchor proteins to demonstrate the feasibility of the two split-GFP assembly systems. To measure the display efficiency of functional proteins, laccase expression was measured using the split-GFP assembly system by co-displaying GFP11 or GFP10-11 tags, respectively.

**Conclusions:**

Our study provides two split-GFP based methods that enable qualitative and quantitative analyses of individual cell display efficiency with a simple workflow, thus facilitating further comprehensive investigations into microbial cell surface display technology. Both split-GFP assembly systems offer a one-step procedure with minimal cost, simplifying the fluorescence analysis of surface-displaying cells.

**Supplementary Information:**

The online version contains supplementary material available at 10.1186/s12934-024-02386-1.

## Introduction

Bacterial surface display enables researchers to immobilize the protein of interest (passenger protein) on cell surfaces, endowing the host cell with specific functions [1]. This approach has been applied in a large variety of fields, such as biocatalysis [2, 3], development of biosensors [4], vaccines [5], antimicrobials [6], protein evolution [7, 8], biosorption of metal ions [9, 10], bioremediation [11], and biorefinery applications [12]. Surface display makes the passenger protein readily accessible to externally introduced substrates, bypassing the membrane barrier [13]. Furthermore, in enzymatic reactions, the cell envelope stabilizes the surface-displayed enzymes, rendering them less susceptible to proteolytic degradation and inactivation in an unfavorable extracellular environment [14]. Bacterial surface display also serves as an efficient method for enzyme immobilization, eliminating the need for protein purification for in vitro catalysis [15, 16].

Prior to utilizing the modified cells for specific applications, it is crucial to verify the successful display of passenger protein on cell surface rather than intracellular expression. A range of fluorescence-based strategies have been employed for the visualization of the displayed protein (Table S1). Initially, the localization of the passenger protein is verified through the use of a specific passenger protein antibody coupled with a fluorescently labeled secondary antibody [17, 18]. This approach provides a precise estimation of surface-displayed protein abundance. However, its practical application is limited due to the requirement for a specific antibody targeting the passenger protein. Even upon identification of a specific antibody protein, its purification time (weeks to months) and cost remain high. Another approach involves fusing small tags, such as 6×His [3, 4], Flag [19], Myc [20–22] and ACP [22], with passenger proteins to establish a universal methodology for visualizing displayed proteins. The primary antibody that specifically targets these tags is subsequently conjugated with fluorescently labeled secondary antibodies, resulting in bright fluorescence for visualization. The key advantage of this approach is that it avoids the requirement for a specific antibody against the passenger protein. Nonetheless, both types of fluorescence immunoassays demand costly primary and secondary antibodies, often necessitating several weeks or even months for antibody generation via animal immunization [23]. Moreover, multiple washing steps are typically required during the incubation process to eliminate nonspecifically adsorbed antibodies, the procedure that often takes several hours at low temperature. An innovative alternative was proposed by Wendel et al. [1], who developed a methodology based on GFP nanobody to characterize the display of the passenger protein. Specifically, a nanobody [24, 25] that exhibits high specificity for GFP was fused with the passenger protein. Successful display was then confirmed by visualizing the fluorescence resulting from incubation with externally added GFP protein [1]. This approach demonstrated a low-cost and simple method to obtain supplementary GFP protein, while the large size of GFP nanobody (111 aa) may potentially influence the expression of passenger proteins in the surface display system. Alternatively, direct fusion of a fluorescent protein with the passenger proteins generates intrinsic fluorescence [21, 26, 27]. In this scenario, no supplementary protein was necessary, making it the most cost-effective option. However, fluorescence could also be detected if the fluorescent protein is remained in the cytoplasm or periplasm, thus obscuring accurate passenger protein localization. Therefore, the accuracy of microbial cell surface display assessment remains challenging.

Green fluorescent protein (GFP) is composed of eleven β-strands with a central helix that accommodates the chromophore [28]. Notably, the superfolder GFP variant can be split between the 10th and 11th β-strands, yielding GFP1-10 (∼25 kDa) and GFP11 (∼1.8 kDa) fragments [29]. While both GFP1-10 and GFP11 fragments are individually non-fluorescent, they spontaneously assemble and emit fluorescence when brought together through complementation [30]. Premature chromophore formation can also take place prior to self-assembly when the superfolder GFP is split into GFP1-9 and GFP10-11 fragments. Through the introduction of a protease cleavage site between the 10th and 11th β-strands or the 9th and 10th β-strands, GFP1-10 [31] or GFP1-9 [32] with a pre-matured chromophore can be obtained by cleaving the superfolder GFP using a protease. Similarly, the pre-matured GFP1-10 or GFP1-9 can rapidly complement GFP11 or GFP10-11, leading to the generation of a fluorescence signal [32]. These self-assembled GFP fragments have been widely applied in various fields, including the analysis of protein topology and subcellular localization [33], investigations into protein solubility [34]. Jiang et al. [32] employed the split-GFP (GFP1-9/GFP10-11) for qualitative imaging of cell surface labelling of G protein-coupled receptors, but the number of displayed proteins was not quantified.

In this study, we proposed two split-GFP-based approaches, denoted as the GFP1-10/GFP11 and GFP1-9/GFP10-11 assembly systems, for the validation of successful display of passenger proteins and the quantification of their abundance. Specifically, the small fragment GFP11 (or GFP10-11) was fused to passenger proteins (e.g. Small ubiquitin-like modifier (SUMO), ubiquitin, and laccase, respectively), and subsequently displayed using different anchor proteins (InaZ, OmpC, and OmpA). The presence or absence of displayed passenger proteins was determined by assessing the fluorescence signal after incubating cells with fragment needed for complementation (GFP1-10 or GFP1-9). To quantify the display efficiency of various anchor proteins, the proportion of positively displayed cells was analyzed using flow cytometry. By establishing an in vitro standard curve, the quantity of passenger proteins displayed on individual cells was successfully calculated. In summary, we present two methods for measuring the efficiency of bacterial surface display, offering notable advantages in terms of simplicity and cost-effectiveness.

## Materials and methods

### Strains and culture conditions

*Escherichia coli* (*E. coli*) DH5α was used for the construction and propagation of plasmids. *E. coli* BL21(DE3) was utilized for the expression and proteins display. *E. coli* strains were cultivated at 37 °C in Luria-Bertani medium (5 g/L yeast extract, 10 g/L tryptone, and 10 g/L NaCl) with or without 50 µg/mL of the indicated antibiotics as required. For LB plates, agar was added to the LB medium at a concentration of 20 g/L.

### Plasmid construction

All the genes and primers employed in this study are listed in Tables S2 and S3. For the overexpression of GFP1-10, GFP1-9, SUMO-GFP11, and ubiquitin-GFP10-11 proteins, pET28a was used as the backbone vector, while *E. coli* BL21(DE3) was utilized as the host strain for protein expression. A 6×His tag was affixed to the C or N terminus of the split-GFP fragments to facilitate protein purification. The pCDFDuet-1 plasmid was used as the backbone vector for surface display. OmpA, OmpC and InaZ were used as anchor proteins, while SUMO-GFP11 and ubiquitin-GFP10-11 were employed as passener proteins to verify the split-GFP assembly. To investigate the display of functional proteins, the laccase gene *CotA* amplified from the genomic DNA of *Bacillus subtilis* 168 was displayed with GFP11 and GFP10-11 tags. The details of plasmid construction are described in the supplementary file.

### Protein overexpression and display

*E. coli* BL21(DE3) strains harboring the pET28a-GFP1-10, pET28a-SUMO-GFP11, pET28a-GFP1-9-3C-10-3C-11 and pET24a-ubiquitin-GFP10-11 plasmids were used for the overexpression of GFP1-10, SUMO-GFP11, GFP1-9 and ubiquitin-GFP10-11 proteins, respectively. Specifically, the *E.coli* BL21(DE3) harboring the pET28a-GFP1-9-3C-10-3C-11 plasmid was able to overexperess mature GFP containing two protease cleavage sites between strands 9, 10 and 11. After obtaining matured GFP, it was cleaved by protease into the three fragments: GFP1-9, GFP10 and GFP11. The GFP1-9 protein with pre-matured fluorophore was then obtained after a seconded purification of the cleaved GFP. The detailed procedure for protein expression and purification is elucidated in the supplementary file, while the SDS-PAGE analysis of the purified proteins can be observed in Fig. S1.

The surface-displayed *E. coli* strains were cultivated in LB medium with 50 µg/mL streptomycin. The display of passenger proteins was induced by the addition of 1 mM IPTG when the culture reached an OD_600_ of 0.6. After incubation in a shaker at 18 °C for 20 h, the cells were harvested and washed with PBS buffer (pH 7.4) via centrifugation at 6000 rpm for 10 min.

### In vitro Split-GFP complementation

For in vitro assembly of GFP1-10 and GFP11 fragments, 100 µL volume of SUMO-GFP11 (0.1 µM) was mixed with an equal volume of GFP1-10 protein at various concentrations (0.05, 0.1, 0.2, 0.4, 0.6, 0.8, 1.0, 2.0, 3.0, 4.0, 10.0, 20.0, 30.0 µM). The mixtures were incubated at 30 °C with shaking at 220 rpm for 12 h. The fluorescenc was recorded (λexc = 488 nm, λem = 530 nm) using an microplate reader (Tecan Infinite 200 Pro). To optimize the incubation time, a 100 µL of SUMO-GFP11 (0.1 µM) was mixed with 100 µL of GFP1-10 protein (4 µM), and the fluorescence intensity was measured every 30 min over a total duration of 10 h.

For in vitro assembly of GFP1-9 and GFP10-11 proteins, 100 µL volume of GFP1-9 (1 µM) was mixed with an equal volume of ubiquitin-GFP10-11 protein at various concentrations (100, 200, 300, 400, 500, 600, 800, 1000, 1500, 2000, 2500, 3000 nM). The mixtures were incubated at 30 °C with shaking 220 rpm for 3 h. The fluorescenc was recorded at λexc = 450 nm and λem = 507 nm. To optimize the incubation time, a 100 µL of ubiquitin-GFP10-11 (1 µM) was mixed with 100 µL of GFP1-9 protein (1 µM), and the fluorescence intensity was recorded at intervals of 20 s over a total duration of 1 h.

### Fluorescence measurement and observation of surface-displaying bacteria

The *E. coli* strains displaying SUMO or laccase with GFP11 tag were named OmpA-SUMO-GFP11, OmpC-SUMO-GFP11, InaZ-SUMO-GFP11, OmpA-CotA-GFP11, OmpC-CotA-GFP11, and InaZ-CotA-GFP11, respectively. The strains were induced using IPTG as described above for passenger protein display. The fluorescence intensity was recorded after adding different concentrations of GFP1-10 protein and incubating for different time intervals. Specifically, to optimize the concentration of GFP1-10 protein, 100 µL samples of displaying cells (OD_600_ = 2.0) were mixed with 100 µL of GFP1-10 protein (ranging from 0.2 µM to 30 µM) in an opaque 96-well microtiter plate. The mixtures were incubated at 30 °C with shaking 220 rpm for 12 h. To determine the optimal incubation time, 100 µL of displaying cells (OD_600_ = 2.0) was mixed with 100 µL of GFP1-10 protein (20 µM), followed by incubation at 30 °C with shaking 220 rpm for 24 h. The fluorescenc was recorded (λexc = 488 nm, λem = 530 nm) using a microplate reader. The fluorescence background was eliminated by subtracting the fluorescence measured when the large GFP segment (GFP1-9 or GFP1-10) was added to the sample (time 0) in each experiment.

The *E.coli* strains displaying ubiqutin or laccase and GFP10-11 tag were abbreviated as OmpA-Ubi-GFP10-11, OmpC-Ubi-GFP10-11, InaZ-Ubiq-GFP10-11, OmpA-CotA-GFP10-11, OmpC-CotA-GFP10-11, and InaZ-CotA-GFP10-11, respectively. The cultivation and incubation conditions were the same to those used for the GFP11 displaying strains in order to optimize the incubation concentration and time, but the fluorescence was measured at λexc = 450 nm and λem = 507 nm [32]. The fluorescence background was eliminated by subtracting the fluorescence measured at time 0 in each experiment. The surface-displaying cells that were incubated with GFP1-10 or GFP1-9 protein were observed using a Leica DM5000B fluorescence microscope (BIOLIGHT, Beijing, China).

### Flow cytometry analysis

The surface-displaying cells that were incubated with GFP1-10 or GFP1-9 protein were diluted with PBS buffer to achieve an OD_600_ of 0.1. Subsequently, the percentage of GFP-positive cells was determined using a FACS MoFlo XDP instrument (Beckman, USA) with an excitation wavelength of 488 nm. A total of 100,000 events were recorded for each sample, and the data were analyzed using FlowJo software (version 10.8.1). The negative control was composed of cells without GFP1-10 or GFP1-9 incubation.

### Quantification of the displayed proteins on individual cell

The number of displayed proteins on individual cell was caculated using the equation:

$${N_{dp}}{\rm{ = }}{{{C_1}{\rm{ \times NA}}} \over {{C_2}{\rm{ \times }}{F_p}}}$$

where *N*_*dp*_ represents the number of displayed proteins on individual positive cell. *C*_*1*_ denotes the concentration of GFP11 or GFP10-11 protein (mol/L), which is determined by converting the fluorescence in the system to protein concentration using in vitro standard curve [35]. *C*_*2*_ denotes the concentration of cells in the system (cells/L), which is determined by using a microscope cell counting chamber. When measuring the amount of protein displayed on individual *E. coli* cell in this study, the cell concentration (*C*_*2*_) was controlled within the range of 10^12^ − 10^13^ cells/L (equivalent to OD_600_ = 1) to reduce the influence of cell scattering on the fluorescence signal. $\text{NA}$ is the Avogardo number (6.02 × 10^23^). ${F}_{p}$ represents the fraction of positively displaying cells, determined by flow cytometry analysis.

### Laccase activity assay

The enzymatic activity of the surface-displayed laccase was assessed by monitoring the oxidation rate of 2,2-azinobis-3-ethylbenzothiazoline-6-sulfonate (ABTS). For the whole-cell catalytic activity assay, the reaction system comprised 10 µL of cells displaying the enzyme (OD_600_ = 20), 20 µL of 1.0 mM ABTS, and 170 µL of 0.1 M sodium acetate buffer (pH 4.0). The absorbance of the reaction mixture was measured at 420 nm using a UV spectrophotometer (Eppendorf, Germany) at 15-second intervals for a duration of 5 min. Enzyme activity was quantified in units (U), defined as the amount of enzyme necessary to catalyze the conversion of 1 µmol substrate per minute.

## Results

### Visualization of surface-displayed proteins via GFP1-10/GFP11 assembly

**Fig. 1:**
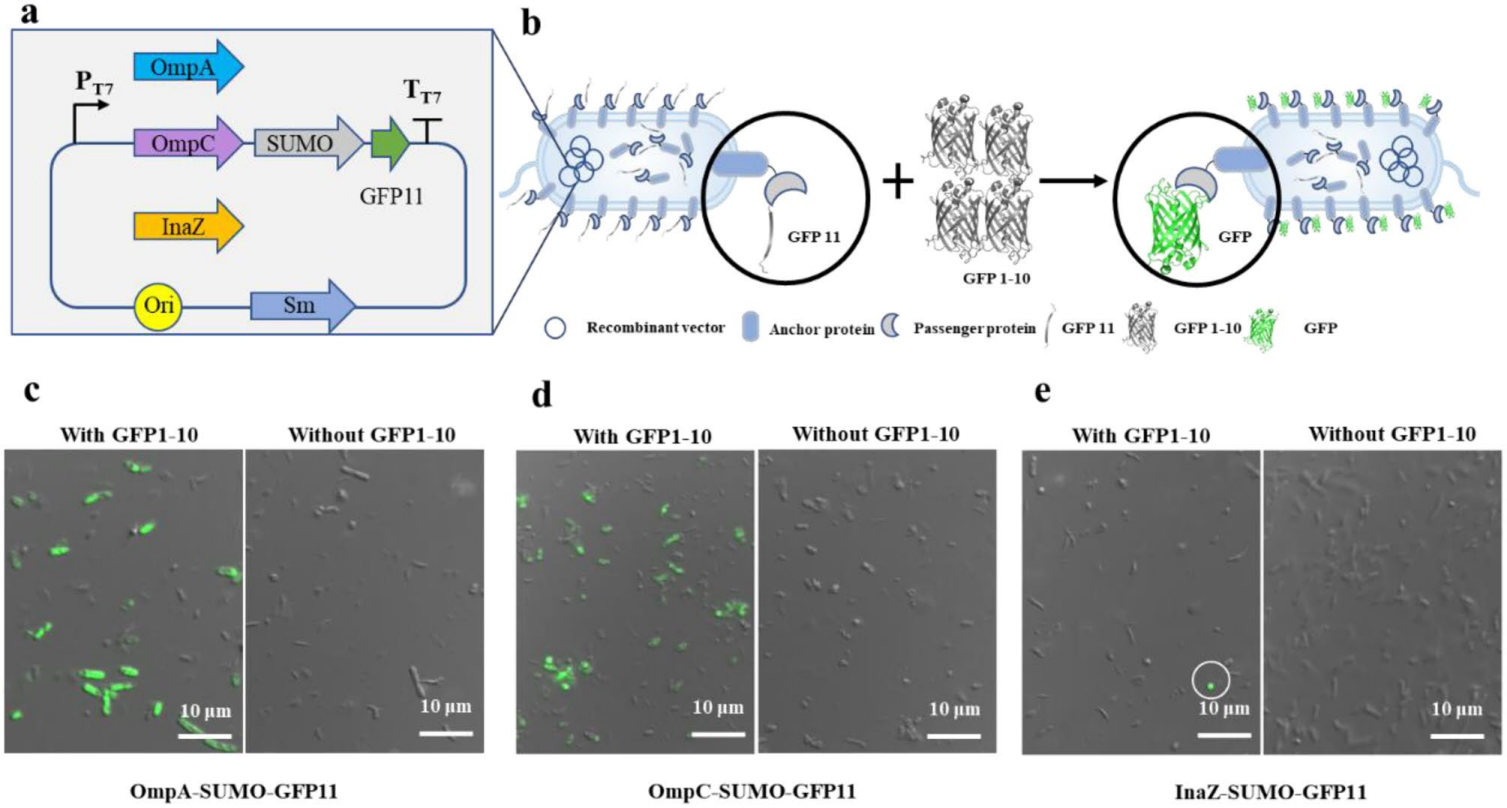
Visualization of cell-surface displayed cells utilizing GFP 1–10/GFP11 assembly. (**a**) Key constituents of plasmids used in the OmpA-SUMO-GFP11, OmpC-SUMO-GFP11, and InaZ-SUMO-GFP11 strains; (**b**) Schematic representation elucidating the assembly of GFP1-10 and GFP11 on the bacterial cell surface; (**c**-**e**) The overlap of bright field images and corresponding fluorescence images of cells displaying OmpA-SUMO-GFP11 (**c**), OmpC-SUMO-GFP11 (**d**), and InaZ-SUMO-GFP11 (**e**), respectively, incubated with (and without) GFP1-10 protein

The primary objective of cell surface display technology is to confirm the successful display of the passenger protein. The spontaneous interaction of fragments GFP1-10 and GFP11, fused to the passenger protein, resulted in the formation of the fluorophore, yielding detectable fluorescence signals (Fig. 1a, b). Within the split-GFP system, the large protein fragment GFP1-10, serving as supplementary component, was unable to traverse the cell membrane. Consequently, the detected fluorescence is exclusively from the assembled GFP at the cell surface.

The GFP11 tag was paired with a solubility enhancer - SUMO, serving as the target protein, while outer membrane protein constituents, namely OmpA [36], OmpC [37], and InaZ [38], were employed as anchors. The efficacy of passenger protein (SUMO-GFP11) display on various anchor proteins was assessed via fluorescence microscopy. Bright green fluorescence was observed in a portion of the OmpA-SUMO-GFP11 cells (Fig. 1c) and OmpC-SUMO-GFP11 cells (Fig. 1d), although a fraction of cells remained non-fluorescent. In the case of InaZ-SUMO-GFP11 cells, the incidence of fluorescent cells was notably lower in comparison to the other two surface-display systems (Fig. 1e), indicating a diminished display efficiency of the InaZ anchor protein. As anticipated, cells incubated without GFP1-10 exhibited no fluorescence signal (Fig. 1c-e).

### Quantification of surface-displayed SUMO via GFP1-10/GFP11 assembly

To quantify the displayed protein on the surface of *E. coli*, an in vitro experiment was performed to evaluate the spontaneous assembly dynamics between GFP11 and GFP1-10 proteins. Purified SUMO-GFP11 and GFP1-10 proteins were co-incubated to initiate the assembly of functional GFP in vitro. The incubation conditions were optimized through systematic manipulation of the molar ratio between GFP1-10 and SUMO-GFP11, as well as the duration of the incubation period. Notably, the fluorescence signal increased significantly with the addition of excess GFP1-10 fragments, reaching a maximum at a molar ratio of 40:1 after a 12-hour incubation period (Fig. S2a). The fluorescence signal demonstrated robust stability, exhibiting no discernible changes even upon successive additions of extra GFP1-10 fragments. Equilibrium in the assembly of SUMO-GFP11 and GFP1-10 was observed to be achieved following a 6.5-hour incubation period, with subsequent measurements confirming the constancy of fluorescence intensity (Fig. S2b). Furthermore, the ratio of surface-displaying cells to GFP1-10 protein, along with the incubation duration, was also optimized (Fig. S3). Specifically, it was established that a GFP1-10 concentration of 15 µM and an incubation time of 10 h were sufficient for the surface-displaying cells (OD_600_ = 1.0) to attain saturated fluorescence.

**Fig. 2:**
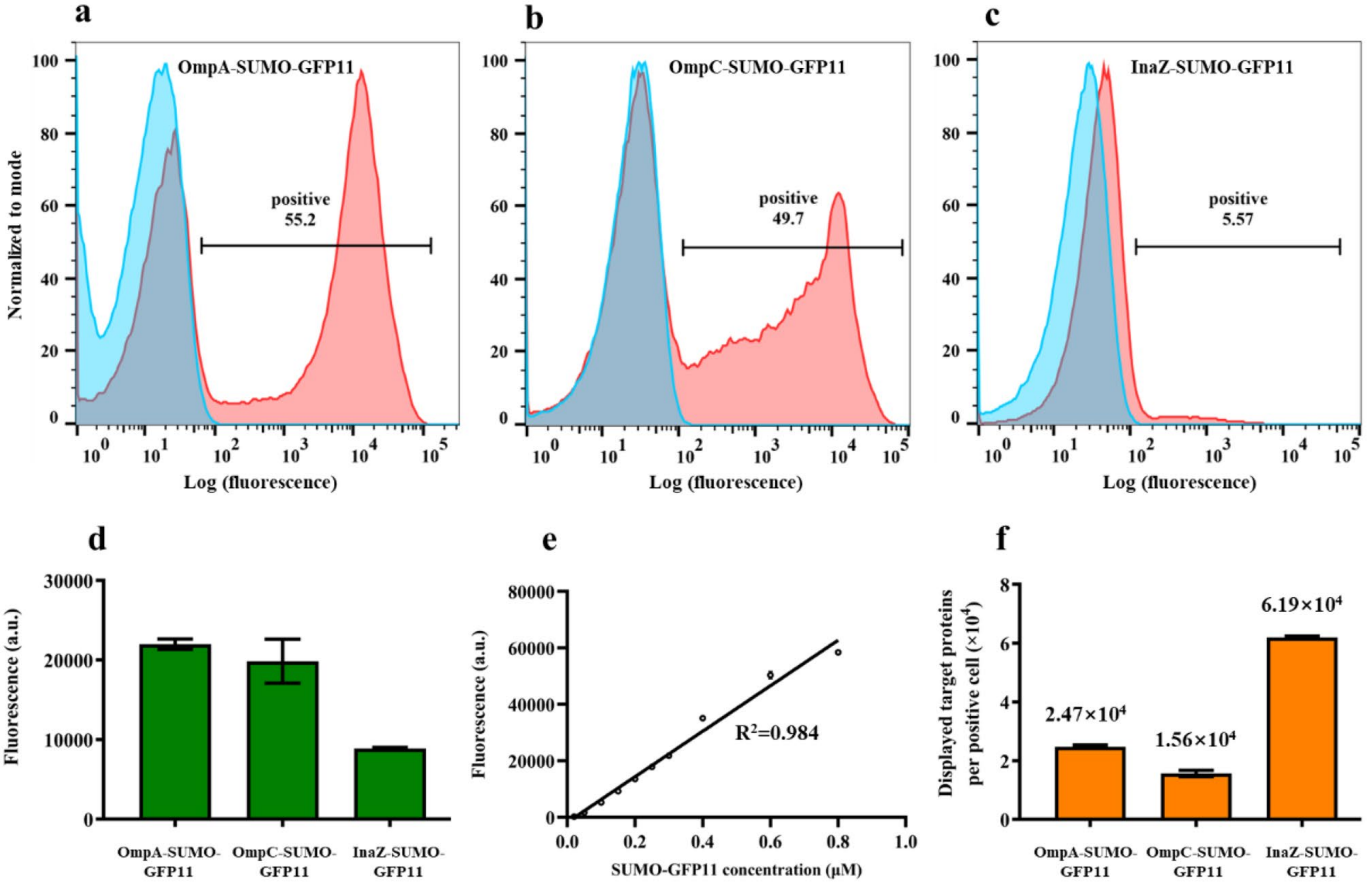
Quantitative assessment of surface-displayed SUMO based on GFP1-10/GFP11 assembly. (**a**-**c**) Flow cytometry analysis of OmpA-SUMO-GFP11, OmpC-SUMO-GFP11, InaZ-SUMO-GFP11 cells incubated with (red) or without (blue) GFP1-10 protein. The percentages of positive fluorescent cells are indicated. (**d**) Whole-cell fluorescence intensity of OmpA-SUMO-GFP11, OmpC-SUMO-GFP11, and InaZ-SUMO-GFP11 cells incubated with GFP1-10 protein measured using a microplate reader. (**e**) In-vitro standard curve for purified SUMO-GFP11 and GFP1-10 assembly. (**f**) Average number of passenger proteins displayed on OmpA-SUMO-GFP11, OmpC-SUMO-GFP11 and InaZ-SUMO-GFP11 cells. The data represent the means ± SD of three independent experiments

Fluorescence imaging revealed discernible variations in the display efficiencies associated with the anchor proteins (OmpA, OmpC, and InaZ). Flow cytometry analysis further clarified that only a fraction of the induced *E. coli* cells effectively displayed the passenger protein (SUMO-GFP11), with the prevalence of displaying cells depending on the particular anchor protein utilized. Specifically, the percentages of positive fluorescent cells were determined to be 55.2%, 49.7%, and 5.57% for OmpA-SUMO-GFP11, OmpC-SUMO-GFP11, and InaZ-SUMO-GFP11, respectively (Fig. 2a-c), aligning with the observations of fluorescence imaging assay in Fig. 1c-e. Furthermore, it is noteworthy that the fluorescence intensity exhibited a broad distribution (ranging from 10^2^ to 10^5^) across all three cell types, indicating the disparate quantities of passenger proteins displayed on the surface of each cell.

The quantification of passenger proteins displayed on individual positive cells utilizing anchor proteins OmpA, OmpC, and InaZ was carried out by employing whole-cell fluorescence analysis in conjunction with a standard curve generated through in vitro experimentation. Initially, the fluorescence intensity of surface-displaying cells was measured using a microplate reader after incubation with GFP1-10 protein (Fig. 2d). Subsequently, a standard curve was established to capture the in vitro assembly dynamics between GFP1-10 and SUMO-GFP11 under optimal incubation conditions (Fig. 2e). Notably, the fluorescence intensity at equilibrium demonstrated a linear correlation with the concentration of SUMO-GFP11, particularly subjected to saturated GFP1-10 incubation (at a ratio of 40:1) (Fig. S2a). A remarkable linear correlation coefficient (R^2^ = 0.984) was observed for SUMO-GFP11 concentrations up to 0.8 µM. The average number of passenger proteins showcased by a single positive *E. coli* cell was determined to be 2.42 × 10^4^, 1.57 × 10^4^, and 6.19 × 10^4^ for OmpA-SUMO-GFP11, OmpC-SUMO-GFP11, and InaZ-SUMO-GFP11, respectively. The high number of passenger proteins displayed on individual cells using InaZ as the anchor protein contributed to the low display efficiency, which was only 5.57%, compared to 55.2% for OmpA and 49.7% for OmpC anchors, respectively. Combining the whole-cell fluorescence intensity results (Fig. 2d), it is observed that the InaZ-SUMO-GFP11 cells exhibited the lowest fluorescence, indicating the lowest total amount of displayed protein, despite having the highest average number of displayed passenger proteins. These results underscore the suitability and reliability of the GFP1-10/GFP11 assembly as a method for the quantitative assessment of surface-displayed proteins.

### Quantification of surface-displayed laccase based on GFP1-10/GFP11 assembly

**Fig. 3:**
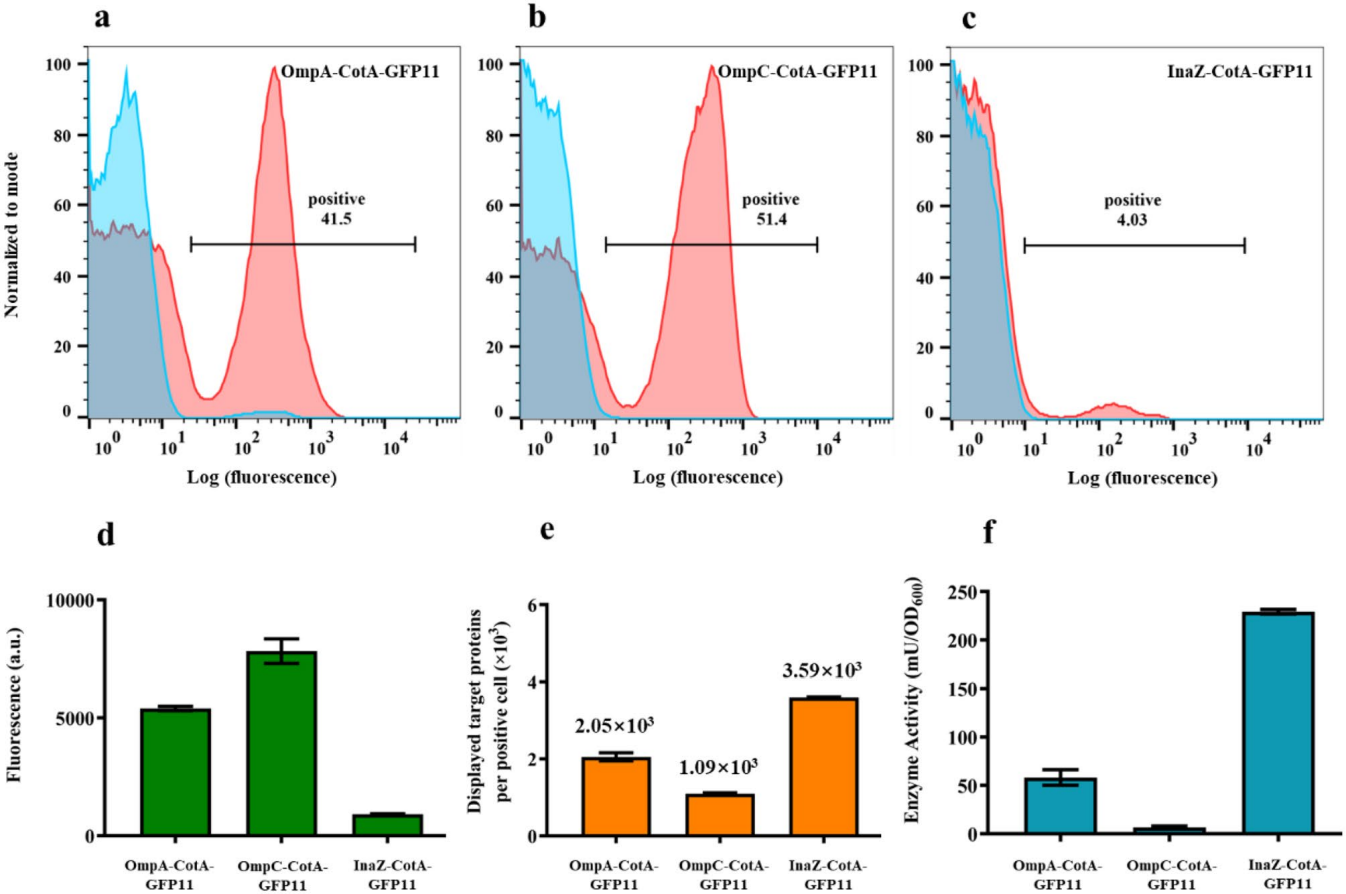
Quantitative measurement of surface-displayed laccase based on GFP1-10/GFP11 assembly. (**a**-**c**) Flow cytometry analysis of OmpA-CotA-GFP11, OmpC-CotA-GFP11, and InaZ-CotA-GFP11 incubated with (red) or without (blue) GFP1-10 protein. (**d**) Whole-cell fluorescence intensity of OmpA-CotA-GFP11, OmpC-CotA-GFP11, InaZ-CotA-GFP11 cells incubated with GFP1-10 protein. (**e**) Average numbers of CotA-GFP11 proteins displayed on OmpA-CotA-GFP11, OmpC-CotA-GFP11, InaZ-CotA-GFP11 cells. (**f**) Laccase activity of OmpA-CotA-GFP11, OmpC-CotA-GFP11, and InaZ-CotA-GFP11 cells. The data represent the means ± SD of three independent experiments

To validate the GFP1-10 and GFP11 assembly method with a functional enzyme, laccase tagged with GFP11 (CotA-GFP11) was displayed on *E. coli* using anchor proteins OmpA, OmpC, and InaZ, respectively, and the number of laccase molecules displayed on individual positive cells was quantitatively determined. Similar to the display of SUMO-GFP11, the percentage of positive cells displaying CotA-GFP11 varied depending on the utilized anchor proteins (Fig. 3a-c), ranging from only 4.03% for InaZ-CotA-GFP11, compared to 41.5% for OmpA-CotA-GFP11, and 51.4% for OmpC-CotA-GFP11.

To quantify the number of passenger proteins displayed on a single cell using OmpA, OmpC, and InaZ, whole-cell fluorescence was measured (Fig. 3d). Combined with the percentages of positive cells, the quantities of CotA-GFP11 proteins displayed on cells using OmpA-CotA-GFP11, OmpC-CotA-GFP11, and InaZ-CotA-GFP11 were determined to be 2.05 × 10^3^, 1.09 × 10^3^, and 3.59 × 10^3^ proteins per positive cell (Fig. 3e). The catalytic performance of the whole cells was further investigated to confirm the functional display of laccase. The enzymatic activities of OmpA-CotA-GFP11, OmpC-CotA-GFP11, and InaZ-CotA-GFP11 cells were determined to be 50.5, 6.9, and 228.6 mU/OD_600_, respectively (Fig. 3f).

Additionally, OD_600_ readings for OmpA-CotA-GFP11, OmpC-CotA-GFP11, and InaZ-CotA-GFP11 after induction for 20 h were 0.98, 1.25, and 3.07, respectively, compared to 3.25 for uninduced cells. Unlike the dispersed property observed in InaZ-CotA-GFP11 and uninduced cells, the OmpA-CotA-GFP11 and OmpC-CotA-GFP11 cells showed clumpy and sticky characteristics. These phenomena indicate growth arrest following IPTG induction when OmpA and OmpC were employed as anchor proteins for laccase display, whereas InaZ-CotA-GFP11 cells exhibited normal bacterial growth. Nanudorn et al. reported a similar result that Lpp-OmpA-ASTB cells had an adverse effect on cell growth [39].

### Visualization of surface-displaying cells via GFP 1–9/ GFP 10–11 assembly

**Fig. 4:**
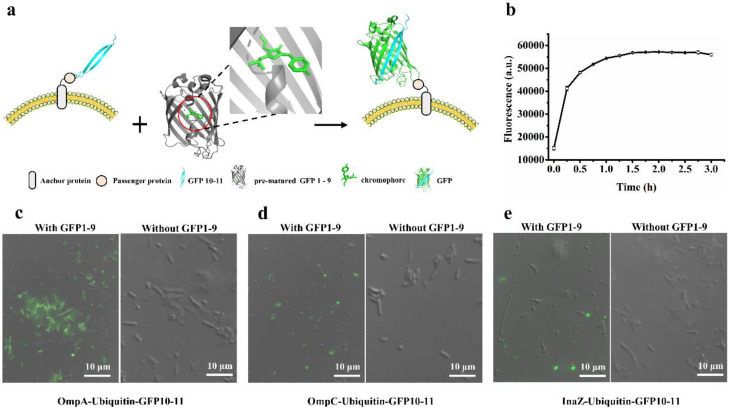
Visualization of surface-displaying cells based on GFP1-9/GFP10-11 assembly. (**a**) Schematic diagram for the assembly of GFP1-9 and GFP10-11 on the bacterial cell surface; (**b**) Time-profile of the fluorescence intensity during assembly of GFP1-9 with surface displaying OmpA-Ubi-GFP10-11 cells. (**c**-**e**) The overlap of bright field images and corresponding fluorescence images of cells displaying OmpA-Ubi-GFP10-11 (**c**), OmpC-Ubi-GFP10-11 (**d**), and InaZ-Ubi-GFP10-11 (**e**), respectively, incubated with (and without) GFP1-9 protein

While GFP1-10/GFP11 assembly system enables both qualitative and quantitative analysis of displayed proteins on the bacterial surface, it is noteworthy that it requires up to 6.5 h (in vitro) to reach equilibrium for fluorescence intensity. As described above, full-length GFP can be split into two fragments for subsequent self-assembly: GFP1-10 and GFP11, or alternatively GFP1-9 and GFP10-11. In the latter case, pre-matured GFP1-9 with a chromophore, derived from intact GFP, assembles efficiently with GFP10-11 within a short time [32]. To evaluate the effectiveness of the split GFP1-9/GFP10-11 assembly system, the solubility enhancer – ubiquitin and GFP10-11 tag was co-displayed using various anchor proteins (OmpA, OmpC, and InaZ). The different displayed *E. coli* cells were then incubated with purified GFP1-9 protein (Fig. 4a). Upon incubation of OmpA-Ubi-GFP10-11 cells with 1µM GFP 1–9 protein, fluorescence saturation was achieved within one hour (Fig. 4b and Fig. S5a-c). Notably, this duration represents only 1/10 of the time required for the OmpA-SUMO-GFP11 cells using the GFP1-10/11 assembly system. Similarly, the incubation periods for OmpC-Ubi-GFP10-11 and InaZ-Ubi-GFP10-11 were determined to be 3 h and 4 h, respectively (Fig. S5), both shorter than their counterparts with the GFP1-10/11 assembly system. Fluorescent imaging of the displayed cells revealed detectable fluorescence in some cells after GFP1-9 incubation, while some others remained non-fluorescent (Fig. 4c-e). The proportion of fluorescent cells using different anchor proteins followed the order of OmpA > OmpC > InaZ. These results indicate that utilizing the self-assembly of GFP1-9/GFP10-11 is an effective mean to promptly verify the surface display of the passenger protein on *E. coli*. The shorter incubation period underscores the efficiency of this method in confirming protein display on the bacterial surface.

### Quantification of surface-displayed ubiquitin via GFP1-9/GFP10-11 assembly

Prior to flow cytometry analysis, we optimized the ratio of GFP1-9 to ubiquitin-GFP10-11 proteins and the in vitro incubation time (Fig. S4). The results revealed that the maximum fluorescence intensity of assembled GFP was achieved when the ubiquitin-GFP10-11 and GFP1-9 proteins were present in a 1:1 ratio and incubated for 0.5 h. Subsequently, we optimized the ratio and incubation time of surface-displaying cells and GFP1-9 protein (Fig. S5). Our findings indicated that a 1 µM GFP1-9 protein and an incubation time of 3 h were sufficient for cells with a concentration equivalent to OD_600_ = 1 to reach fluorescence equilibrium.

**Fig. 5:**
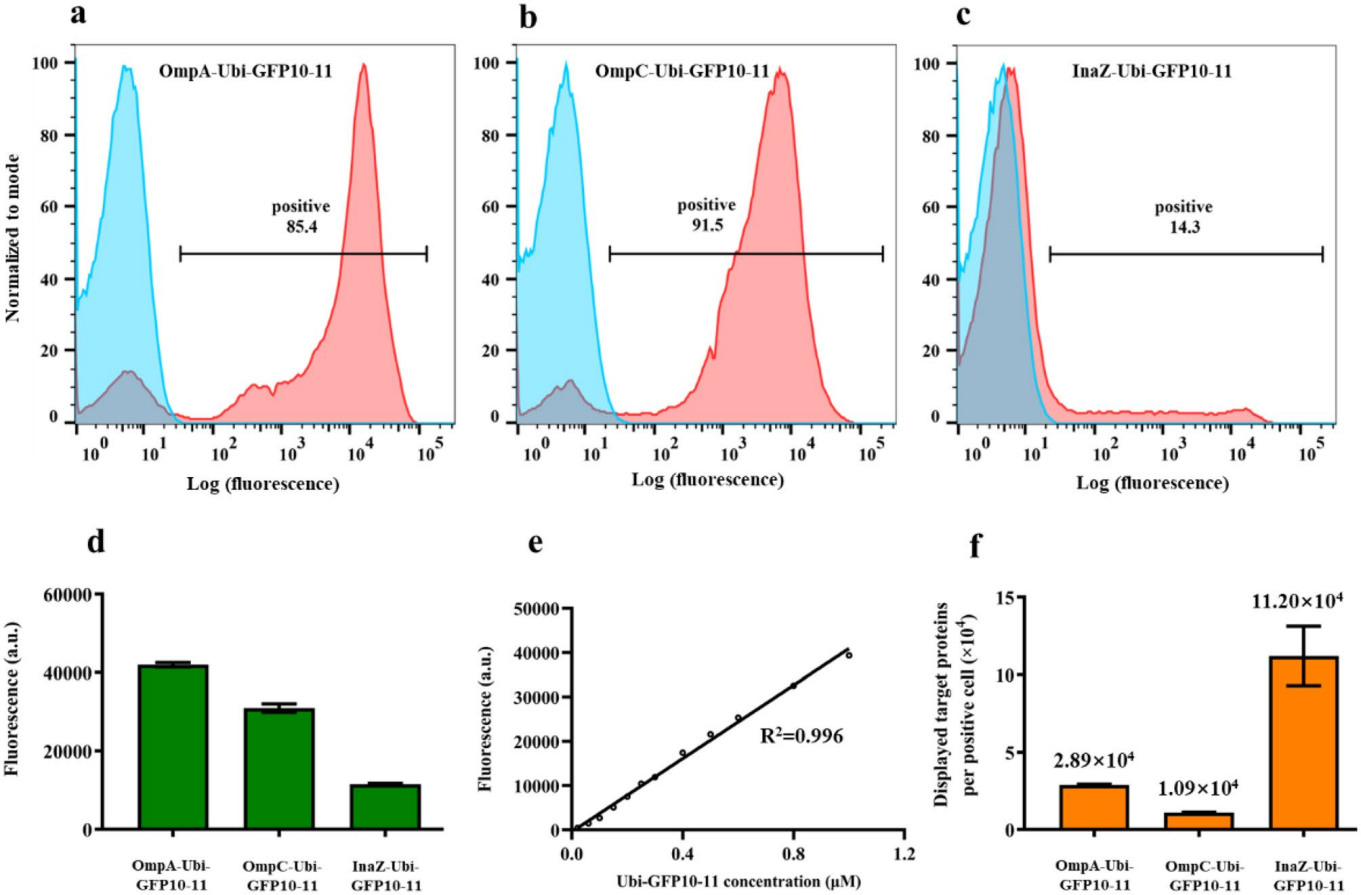
Quantitative measurement of surface-displayed ubiquitin using GFP1-9/GFP10-11 assembly. (*a*-*c*) Flow cytometry analysis of OmpA-Ubi-GFP10-11, OmpC-Ubi-GFP10-11, and InaZ-Ubi-GFP10-11 incubated with (red) or without (blue) GFP1-9. The percentages of positive fluorescent cells are indicated. (*d*) Whole cell fluorescence intensity of OmpA-Ubi-GFP10-11, OmpC-Ubi-GFP10-11, and InaZ-Ubi-GFP10-11 cells incubated with GFP1-9 protein measured using a microplate reader. (*e*) Standard curve for purified ubiquitin-GFP10-11 and GFP1-9 assembly *in-vitro*. (*f*) Average numbers of passenger proteins displayed on OmpA-Ubi-GFP10-11, OmpC-Ubi-GFP10-11, and InaZ-Ubi-GFP10-11 cells. The data represent the means ± SD of three independent experiments

Flow cytometry analysis revealed that 85.4%, 91.5%, and 14.3% of cells successfully displayed ubiquitin using OmpA-Ubi-GFP10-11, OmpC-Ubi-GFP10-11, and InaZ-Ubi-GFP10-11, respectively (Fig. 5a-c). Specifically, the surface-displayed cells exhibited bimodal distributions in flow cytometry after GFP1-9 incubation: the left peak represented the non-displayed cells, while the right peak indicated the successfully displayed cells. Subsequently, their whole-cell fluorescence levels were measured after incubation with GFP 1–9 protein (Fig. 5d), and then calculated by subtracting the background fluorescence, which was relatively high at a concentration of 1 µM (Figure S6). To determine the number of ubiquitin proteins displayed using each surface display system, we constructed an in vitro standard curve under the optimized incubation conditions. A remarkably high linear correlation (R^2^ = 0.996) was observed between the concentration of ubiqium-GFP10-11 and the fluorescence intensity (Fig. 5e). Utilizing this standard curve, we calculated the number of displayed ubiquitin proteins per positive cell, taking into account the display efficiency. The results (Fig. 5f) showed that 2.89 × 10^4^, 1.09 × 10^4^ and 1.12 × 10^5^ ubiquitin proteins were displayed on each positive cell using OmpA-Ubi-GFP10-11, OmpC-Ubi-GFP10-11, and InaZ-Ubi-GFP10-11, respectively. It appeared that InaZ displayed the highest number of proteins in individual cells; however, the total number of proteins displayed on all cells was the lowest (Fig. 5d) due to InaZ possessing the lowest display efficiency (Fig. 5c).

### Quantification of surface-displayed laccase using GFP 1–9/ GFP10-11 assembly

**Fig. 6:**
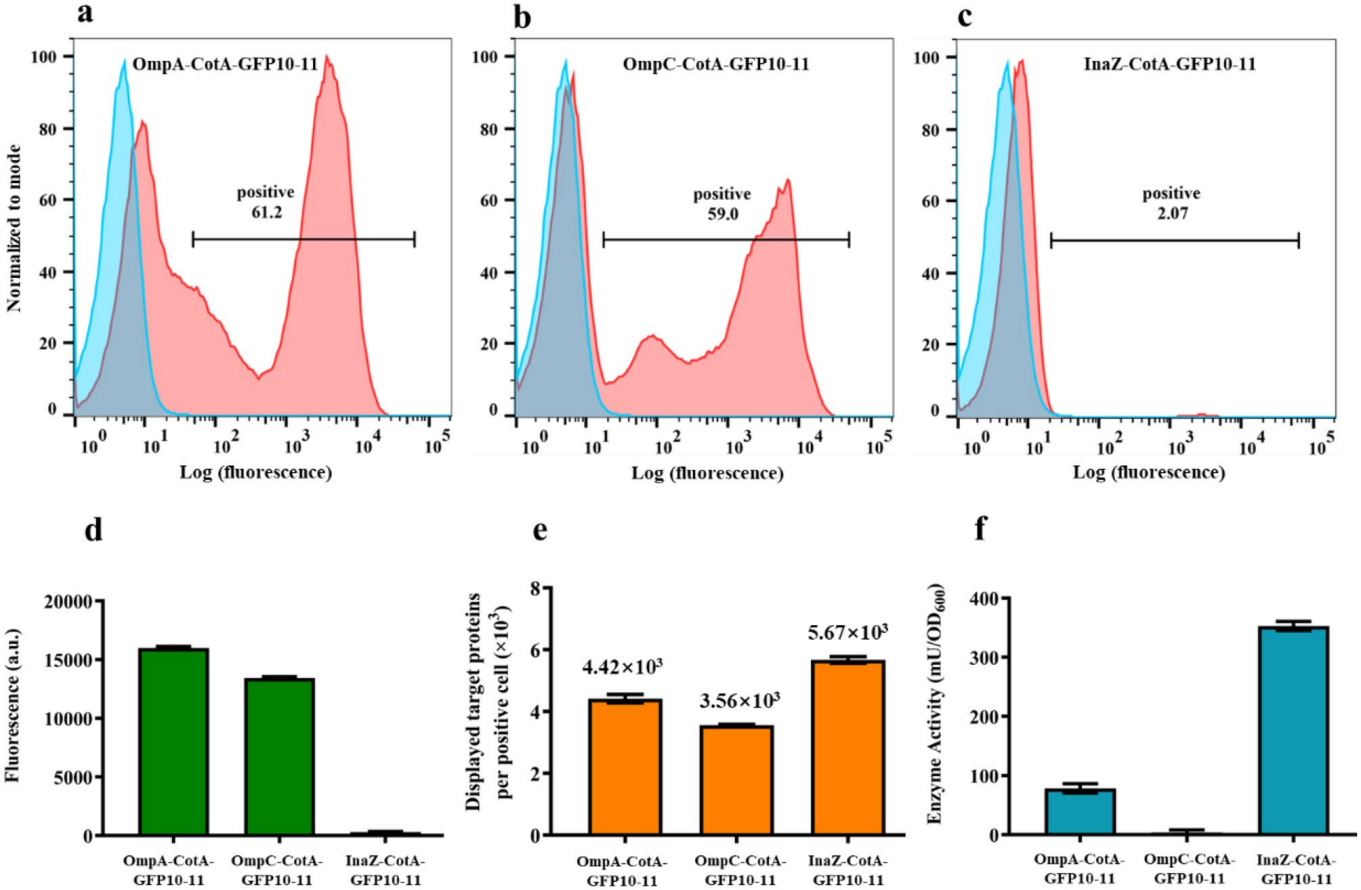
Quantitative measurement of surface-displayed laccase using GFP 1–9/GFP10-11 assembly. (*a*-*c*) Flow cytometry analyses of OmpA-CotA-GFP10-11, OmpC-CotA-GFP10-11, and InaZ-CotA-GFP10-11 cells with (red) or without (blue) GFP1-9. (*d*) Whole cell fluorescence intensity of OmpA-CotA-GFP10-11, OmpC-CotA-GFP10-11, and InaZ-CotA-GFP10-11 cells incubated with GFP1-9 protein. (*e*) Average numbers of CotA-GFP10-11 molecules displayed on OmpA-CotA-GFP10-11, OmpC-CotA-GFP10-11, and InaZ-CotA-GFP10-11 cells. (*f*) Laccase activity of OmpA-CotA-GFP10-11, OmpC-CotA-GFP10-11, and InaZ-CotA-GFP10-11 cells. The data represent the means ± SD of three independent experiments

To assess the number of functional surface-displayed laccase enzymes, we employed the GFP1-9/GFP10-11 assembly system. FACS analyses revealed successful laccase display on a subpopulation of the OmpA-CotA-GFP10-11, OmpC-CotA-GFP10-11, and InaZ-CotA-GFP10-11 cells, with proportions of 61.2%, 59%, and 2.07%, respectively (Fig. 6a-c). Furthermore, fluorescence intensities were measured to quantify the number of surface-displayed laccase molecules (Fig. 6d). After calculation, the numbers of laccase molecules displayed on each positive cell using OmpA-CotA-GFP10-11, OmpC-CotA-GFP10-11, and InaZ-CotA-GFP10-11 were 4.42 × 10^3^, 3.56 × 10^3^, and 5.67 × 10^3^, respectively (Fig. 6e). Finally, the enzymatic activities of all three strains were measured as well, and were found to reach 72.9, 1.2, and 347.4 mU/OD_600_, respectively (Fig. 6f). Similar to the display of laccase with the GFP11 tag using OmpA, OmpC, and InaZ as anchor proteins, growth cessation was observed for the OmpA-CotA-GFP10-11 and OmpC-CotA-GFP10-11 cells, while InaZ-CotA-GFP10-11 cells exhibited normal growth.

## Discussion

Microbial cell surface display has emerged as a promising approach to expose passenger proteins on the surface of microbial cells, endowing them to acquire specific functionalities. The functional efficacy of passenger proteins in surface display is influenced by three critical factors: (1) the successful display of passenger proteins on the cell surface, (2) the efficiency of the employed anchor proteins in facilitating display, and (3) the quantification of displayed proteins on individual cells.

This study presents two innovative methods based on split-GFP assembly for the quantitative determination of proteins displayed on microbial cell surface. Two split-GFP systems, namely GFP1-10/GFP11 and GFP1-9/GFP10-11, were employed to validate the localization of passenger proteins in the surface display system. Given the challenge of the introduced GFP1-10 (GFP1-9) proteins to penetrate the cell membrane, the observed fluorescence on *E. coli* cells originates from the assembly of GFP11 with GFP1-10 (GFP10-11 with GFP1-9) on the cell surface [40]. OmpA, OmpC, and InaZ have been previously reported as efficient anchor proteins for *E. coli* surface display [41, 42]. Consequently, these proteins were selected as anchors in our study. Small proteins, namely SUMO and ubiquitin, were fused with GFP11 or GFP10-11 tags to demonstrate the feasibility of split-GFP assembly for surface display. The observation of bright fluorescence complementation, resulting from the reassembly of the split-GFP fragments, conclusively validated the functional display of passenger proteins. However, it is essential to highlight that fluorescence was not observed in all cells, suggesting that the display efficiency of these anchor proteins fell short of 100% [40].

Accurately quantifying the number of passenger proteins displayed on individual cells is a critical aspect in the assessment of surface display techniques. Conventionally, immunoassays and western blotting were used to quantify displayed proteins. For example, it has been reported that approximately 6 × 10^4^ single-chain Fv antibody fragments were affixed to the surface of *E. coli* [43]. In *Bacillus subtilis*, the number of cell wall-binding modules was assessed through western blotting, revealing that each filamentous cell could display 1.1 × 10^8^ β-lactamase molecules [44]. In our study, we utilized the split-GFP system to quantify the number of displayed passenger proteins. Following the incubation of cells with GFP1-10 or GFP1-9 fragments, the subsequent rise in fluorescence intensity was compared to an in vitro standard fluorescence curve. Our calculations revealed that the number of passenger proteins displayed on *E. coli* cells fell within the range of 10^4^ to 10^5^ when employing OmpA, OmpC, and InaZ as anchor proteins. This observation aligns with prior research findings [9, 43]. Notably, the split-GFP system offers several advantages over traditional immunoassays and western blotting methods, eliminating the requirement for primary and secondary antibodies and consequently reducing experimental costs. Furthermore, the streamlined quantification process renders repeated antibody incubation and washing steps unnecessary.

In surface display technology, the presentation of functional enzymes on cell surfaces holds significant importance in guaranteeing their optimal catalytic performance. In this study, we aimed to qualitatively and quantitatively demonstrate the display of laccase, a protein with a molecular weight of approximately 65 kDa, using both the GFP1-10/GFP11 and GFP1-9/GFP10-11 systems. Consistent with the display of smaller proteins such as SUMO and ubiquitin, our observations indicate that anchor proteins OmpA and OmpC exhibited higher display efficiency compared to the anchor protein InaZ. However, when employing InaZ as the anchor protein, *E. coli* displayed laccase with the highest enzymatic activity. It is noteworthy that the OmpA and OmpC display systems showed a high surface display efficiency but low enzymatic activity. The functionality of laccase is less likely to be influenced by the GPF11 or GFP10-11 tags due to their short length of 16 and 33 amino acids, respectively. The significant inhibition of cell growth may contribute to the observed low enzymatic activity [39, 45]. In our quantitative assessment of passenger proteins through the split-GFP assembly systems, we determined that approximately 10^4^ − 10^5^ small proteins (SUMO and ubiquitin) and 10^3^ laccase molecules were displayed per *E. coli* cell. Importantly, the calculated number of passenger proteins using both the GFP1-10/GFP11 and GFP1-9/GFP10-11 systems fell within the same order of magnitude, indicating the stability and consistency of these two evaluation methods.

Both the GFP1-10/GFP11 and GFP1-9/GFP10-11 assembly systems proved effective in qualitatively and quantitatively assessments of the number of passenger proteins displayed on the cell surface. However, these systems differ in their basic characteristics, protein preparation, and incubation parameters (Table 1). The GFP11 and GFP10-11 tags, consisting of 16 and 33 amino acids, respectively, were used as co-displayed tags on the cell surface, alongside with the passenger protein. These tags do not interfere with the display of passenger proteins, similar to other small tags commonly used in immunofluorescence assays, such as the 6×His, Flag, HA, c-Myc, and GST tags. Notably, the background fluorescence of GFP1-10 was relatively low in comparison to GFP1-9. Despite the overexpression of GFP1-10 resulting in the formation of a substantial amount of inclusion bodies, soluble proteins can be obtained using chemical denaturation and renaturation methods without requiring additional equipment. In comparison to existing methods for verifying the successful display of passenger proteins (Table S1), this remarkable simple method for obtaining GFP1-10 supplementary protein reduces both protein purification costs and operational complexity. In contrast, GFP1-9 protein is derived from full-length GFP through proteolytic cleavage, ensuring that its chromophore is already mature prior to incubation process. The purification process of GFP1-9, which spans approximately 5 days, entails 3 C protease digestion, chemical denaturation, renaturation, and column purification, requiring protein purification equipment (AKTA Purifier). In the incubation process of GFP1-10/GFP11 assembly, the chromophore matures through the reconstitution of the GFP1-10 and GFP11 fragments, involving autocatalytic cyclization, dehydration, and oxidation of three specific amino acid residues (Ser65, Tyr66, and Gly67) [46]. Consequently, achieving saturated fluorescence in *E. coli* cells at a density corresponding to OD_600_ = 1 required a longer incubation time (12 h) and a higher concentration of GFP1-10 protein (15 µM). In contrast, achieving saturated fluorescence in the assembly of GFP1-9/GFP10-11 required only 1 µM of GFP1-9 protein and 3 h of incubation, attributed to the presence of the pre-formed chromophore. The straightforward incubation conditions for split-GFP assembly systems, characterized by a simple incubation process (no requirement for a dark environment) and room temperature incubation, confer advantages over other existing methods (Table S1). Most importantly, both split-GFP assembly systems enable qualitative and quantitative analysis of the displayed protein on individual cell, whereas existing fluorescence-based strategies can only qualitatively visualize the displayed proteins.

**Table 1:** Key characteristics of the GFP1-10/GFP11 and GFP1-9/GFP10-11 assembly systems

	Characteristics	GFP1-10/GFP11	GFP1-9/GFP10-11
Basic information	Tag length	16 a.a.	33 a.a.
	Pre-maturity	No	Yes
	Fluorescence background	Low	High
Supplementary protein preparation	Time required	7 h	5 days
	Purification steps	Simple	Complicated
	Purification equipment	No	Yes
Incubation parameters	Incubation time	12 h	3 h
	Protein added to *E. coli* (OD_600_ = 1)	15 µM	1 µM
Quantification		Yes	Yes

## Conclusions

In this study, we developed two methods for accurately quantifying the number of passenger proteins displayed on *E. coli* utilizing split-GFP technology. The fluorescence of the assembled GFP, comprising surface-displayed SUMO-GFP11 and the supplementary GPF1-10 protein, was visualized to achieve this quantification. To quantitatively calculate the number of displayed SUMO-GFP11 proteins, we first determined the proportions of positive displaying cells using anchor proteins OmpA, OmpC, and InaZ. Through the comparison of the fluorescence with an in vitro standard curve, we were able to determine the number of displayed SUMO-GFP11 proteins. These anchor proteins exhibited distinct characteristics in terms of protein display. For instance, OmpA exhibited the highest display efficiency (∼ 55%), while InaZ displayed the most proteins pre positive cell (6.19 × 10^4^ proteins/cell). As a functional enzyme, laccase was displayed at an order of magnitude lower abundance, approximately10^3^ per cell. The anchor protein InaZ exhibited the lowest display efficiency of 4.03%. However, it displayed 3.59 × 10^3^ laccase per cell, leading to the highest enzyme activity observed at 228.6 mU/OD_600_.

The GFP1-9/GFP10-11 assembly system was also investigated using the same approach. Fluorescent images demonstrated the feasibility of this method. The number of displayed ubiquitin-GFP10-11 and laccase proteins were within the orders of magnitude of 10^3^ to 10^4^, consistent with the results obtained using the GFP1-10/GFP11 system. Interestingly, the highest display efficiency did not necessarily correlate with the highest number of displayed proteins on individual cells or the highest enzyme activity, possibly due to cell death caused by excessively high display efficiency. Thus, for accurate quantification of heterogeneously surface-displayed protein in individual cell, it is crucial to consider the overall display efficiency on a cell population basis. Furthermore, the total number of functionally displayed proteins on living cells, which is affected by display efficiency and the amount of displayed protein on individual cells, is critical factor that influences enzymatic activity. Although both split-GFP assembly systems can be employed for the quantification of displayed proteins, differences exist in their fundamental characteristics, protein preparation and incubation parameters. For example, obtaining pure soluble GFP1-10 protein only requires a simple purification process of 7 h, but achieving saturated fluorescence demands a higher protein concentration of 15 µM and a longer incubation time of 12 h. On the other hand, the purification process for pre-matured GFP1-9 takes 5 days, but 3 hours of incubation with 1 µM protein is sufficient to achieve saturated fluorescence. Both split-GFP assembly systems offer a one-step procedure with minimal cost, simplifying the fluorescence analysis of surface-displaying cells.

### Electronic supplementary material

Below is the link to the electronic supplementary material.

Supplementary Material 1

## Data Availability

No datasets were generated or analysed during the current study.
