# Supplementary material for: Quantitative measurement of cell-surface displayed proteins based on split-GFP assembly
Source: Microb Cell Fact. 2024 Apr 12;23:108. doi: 10.1186/s12934-024-02386-1 (PMC11015686; doi:10.1186/s12934-024-02386-1)
Supplement: Supplementary file 1 — Supplementary Material 1 [file 12934_2024_2386_MOESM1_ESM.docx]

**Supplementary Information for:**

**Quantitative measurement of cell-surface displayed proteins based on split-GFP assembly**

Li Zhang^1, 2#^, Ling Tan^1, 3#^, Meizi Liu^1, 3^, Yunhong Chen^1, 4^, Yu Yang^2*^, Yanfei Zhang^1, 4*^, Guoping Zhao^4, 5^

^1^ Tianjin Institute of Industrial Biotechnology, Chinese Academy of Sciences, Tianjin 300308, China.

^2^ School of Minerals Processing and Bioengineering, Central South University, Changsha, Hunan, 410083 P.R. China

^3^ Haihe Laboratory of Synthetic Biology, Tianjin 300308, China.

^4^ National Center of Technology Innovation for Synthetic Biology, Tianjin 300308, China.

^5^ CAS-Key Laboratory of Synthetic Biology, CAS Center for Excellence in Molecular Plant Sciences, Institute of Plant Physiology and Ecology, Chinese Academy of Sciences, Shanghai, 200032, China.

# These authors contributed equally to this work.

*Correspondence: [zhangyf@tib.cas.cn](mailto:zhangyf@tib.cas.cn) and [csuyangyu@mail.csu.edu.cn](mailto:csuyangyu@mail.csu.edu.cn)

1. **Plasmid construction**

Construction of expression plasmids for *in-vitro* split-GFP assays: For *in-vitro* split-GFP assembly, the genes for GFP1-10^1^, GFP1-9-3C-10-3C-11, SUMO-GFP11 and ubiquitin-GFP10-11 (Tab. S2) were synthesized by Tsingke Biotechnology (Beijing, China). The gene encoding GFP1-10 with a 6His tag at the N terminus was inserted between the *Nco*I and *Xho*I restriction sites of the pET28a vector, resulting in pET28a-GFP1-10. For the expression of SUMO-GFP11, the SUMO-GFP11 gene was inserted between the *Nhe*I and *Sac*I sites of pET28a, resulting in pET28a-SUMO-GFP11 (6 His tag at the N terminal). GFP1-9 was obtained via the hydrolysis of full GFP by 3C protease. The gene encoding GFP1-9-3C-10-3C-11 was inserted between the *Nhe*I and *Xho*I restriction sites of pET28a-GFP1-10, resuting in pET28a-GFP1-9-3C-10-3C-11 (6 His tag at the N terminus). Similarly, the gene encoding ubiquitin-GFP10-11 was inserted between the *Nde*I and *Xho*I sites of pET24a, resulting in pET24a-ubiquitin-GFP10-11 (6 His tag at the C terminus). Subsequently, all plasmids were individually introduced into *E. coli* BL21(DE3) cells to construct strains for the expression of GFP1-10, SUMO-GFP11, GFP1-9-3C-10-3C-11, and ubiquitin-GFP10-11, respectively.

Construction of GFP11 surface display plasmids: All the primers used in this study are listed in Table S3. The pCDFDuet-1 plasmid was digested with *Nde*I and *Xho*I to genereate the pCDFDuet backbone. For the pCDFDuet-Lpp-OmpA-SUMO-GFP11 plasmid, the OmpA fragment amplified by pZL1 and pZL2, the SUMO-GFP11 fragment amplified by pZL3 and pZL4, and the pCDFDuet backbone were ligated by Gibson assembly. Similarly, the OmpC fragment amplified by pZL5 and pZL6, the SUMO-GFP11 fragment amplified by pZL7 and pZL4 were ligated with the pCDFDuet backbone, resulting in the pCDFDuet-OmpC-SUMO-GFP11 plasmid. The InaZ fragment amplified by pZL8 and pZL9, the SUMO-GFP11 fragment amplified by pZL10 and pZL4 were ligated with the pCDFDuet backbone, resulting in the pCDFDuet-InaZ-SUMO-GFP11 plasmid. For the functional display of laccase, the *cotA* gene was amplified from genomic DNA of *Bacillus subtilis* 168 using pZL11 and pZL12. Subsequently, the SUMO coding sequence in pCDFDuet-Lpp-OmpA-SUMO-GFP11, pCDFDuet-OmpC-SUMO-GFP11, and pCDFDuet-InaZ-SUMO-GFP11 was replaced by the *cotA* coding sequence using the primers pZL13 - pZL18, resulting in pCDFDuet-Lpp-OmpA-CotA-GFP11, pCDFDuet-OmpC-CotA-GFP11, and pCDFDuet-InaZ-CotA-GFP11, respectively. The *E. coli* (DE3) strains harboing pCDFDuet-Lpp-OmpA-SUMO-GFP11, pCDFDuet-OmpC-SUMO-GFP11, pCDFDuet-InaZ-SUMO-GFP11, pCDFDuet-Lpp-OmpA-CotA-GFP11, pCDFDuet-OmpC-CotA-GFP11, and pCDFDuet-InaZ-CotA-GFP11 were abbreviated as OmpA-SUMO-GFP11, OmpC-SUMO-GFP11, InaZ-SUMO-GFP11, OmpA-CotA-GFP11, OmpC-CotA-GFP11, and InaZ-CotA-GFP11, respectively.

Construction of GFP10-11 surface display plasmids: the SUMO-GFP11 coding sequence of the pCDFDuet-Lpp-OmpA-SUMO-GFP11, pCDFDuet-OmpC-SUMO-GFP11, and pCDFDuet-InaZ-SUMO-GFP11 plasmids was replaced by ubiquitin-GFP10-11 using primers pZL19 - pZL26, resulting in pCDFDuet-Lpp-OmpA-Ubiquitin-GFP10-11, pCDFDuet-OmpC-Ubiquitin-GFP10-11, and pCDFDuet-InaZ-Ubiquitin-GFP10-11, respectively. Likewise, the primers pZL27 - pZL34 were employed to replace the ubiquitin coding sequence, resulting in the plasmids pCDFDuet-Lpp-OmpA-CotA-GFP10-11, pCDFDuet-OmpC-CotA-GFP10-11 and pCDFDuet-InaZ-CotA-GFP10-11, respectively. The *E. coli* (DE3) strains harbring pCDFDuet-Lpp-OmpA-Ubiquitin-GFP10-11, pCDFDuet-OmpC-Ubiquitin-GFP10-11, pCDFDuet-InaZ-Ubiquitin-GFP10-11, pCDFDuet-Lpp-OmpA-CotA-GFP10-11, pCDFDuet-OmpC-CotA-GFP10-11, and pCDFDuet-InaZ-CotA-GFP10-11 were abbreviated as OmpA-Ubi-GFP10-11, OmpC-Ubi-GFP10-11, InaZ-Ubi-GFP10-11, OmpA-CotA-GFP10-11, OmpC-CotA-GFP10-11, and InaZ-CotA-GFP10-11, respectively.

1. **Protein expression and purification**

**2.1 Purification of GFP 1-10 protein**

**Reagent preparation:**

TNG buffer: 100 mM Tris-HCl (pH 7.4), 150 mM NaCl, 10% glycerol v/v %.

**Purification steps:**

1. **Bacteria inoculation:**

- Inoculate a single colony of *E. coli* BL21(DE3) carrying the pET28a-GFP1-10 plasmid into 10 mL of LB medium supplemented with 50 μg/mL kanamycin.
- Incubate the culture overnight on a shaking table at 37 °C and 220 rpm.

1. **Bacteria culture.**

- Use the overnight culture to inoculate 800 mL of fresh LB medium (inoculation volume of 1%) containing 50 μg/mL kanamycin.
- Incubate the culture at 37 °C with shaking at 220 rpm.
- Add IPTG to a final concentration of 1 mM when the culture reaches an OD600 of 0.6 - 0.8.
- Continue culturing the *E. coli* cells at 37 °C for 5 hours to induce GFP 1-10 inclusion body formation.

1. **Acquisition of GFP1-10 inclusion bodies.**

- Centrifuge the cells at 4500 rpm for 15 minutes to collect them.
- Resuspend the pellet in 15 mL of TNG buffer.
- Lyse the cell suspension with 1 mg/mL lysozyme at 4 °C for 2 hours.
- Further disrupt the cells by ultrasonication on ice for 40 minutes(1 s ON and 3 s OFF).
- Centrifuge the lysate at 12000 rpm and 4 °C for 60 minutes.
- Repeat the above steps twice to obtain GFP1-10 inclusion bodies.

1. **Chemical denaturation of GFP1-10 inclusion bodies.**

- Add a suitable amount of 8 M urea to the pellet to achieve a cell concentration of 75 mg/mL.
- Incubate the mixture at 37 °C until the inclusion bodies completely dissolve.
- Centrifuge the solution at 13000 rpm for 10 minutes to remove insoluble debris.

1. **Renaturation of GFP1-10 protein.**

- Add TNG buffer to the supernatant at a ratio of 25:1.
- Filter the solution through a 0.2 μm pore-size filter membrane.
- Confirm the purity of renatured GFP1-10 in solution by SDS-PAGE.

1. **Determination of GFP1-10 concentration.**

- Measure the concentration using a NanoDrop spectrophotometer at 280 nm.
- Calculate the concentration of the protein sample in mg/mL using the following equation.

$$C_{p}=\frac{A_{280}}{\varepsilon_{0.1\%}}$$

Where $C_{p}$ (mg/mL) is the concentration of protein sample, $A_{280}$ is the measured UV absorbance at 280 nm, $\varepsilon_{0.1\%}$ is the protein extinction coefficient, which can be calculated from amino acid sequence of protein sample (https://www.novoprolabs.com/tools/protein-extinction-coefficient-calculation).

**2.2 Purification of SUMO-GFP11:**

**Reagent preparation:**

Washing buffer 1: 1×PBS (pH=7.4) with10% glycerol and 30 mM imidazole.

Washing buffer 2: 1×PBS (pH=7.4) with10% glycerol and 50 mM imidazole.

Washing buffer 3: 1×PBS (pH=7.4) with10% glycerol and 80 mM imidazole.

Elution buffer: 1×PBS (pH=7.4) with10% glycerol and 200 mM imidazole.

**Purification steps:**

1. **Bacteria inoculation.**

- Inoculate a single colony of *E. coli* BL21(DE3) carrying the pET28a-SUMO-GFP11 plasmid into 10 mL of LB medium supplemented with 50 μg/mL kanamycin.
- Incubate the culture overnight on a shaking table at 37 °C and 220 rpm.

1. **Bacteria culture.**

- Use the overnight culture to inoculate 800 mL of fresh LB medium (inoculation volume of 1%) containing 50 μg/mL kanamycin.
- Incubate the culture at 37 °C with shaking at 220 rpm.
- Add IPTG to a final concentration of 1 mM when the culture reaches an OD600 of 0.6 - 0.8.
- Culture the *E. coli* cells at 30 °C for 12 hours to express SUMO-GFP11.

1. **Acquisition of SUMO-GFP11** **crude extracts.**

- Centrifuge the cells at 4500 rpm for 15 minutes to collect them.
- Resuspend the pellet in 15 mL of binding buffer.
- Ultrasonicate the cell suspension on ice for 40 minutes (1 s ON and 3 s OFF).
- Centrifuge the lysate at 12000 rpm at 4 °C for 60 minutes.

1. **SUMO-GFP11 purification.**

- Incubate the supernatant with Ni-NTA beads for 1 hour at 4°C to reach binding equilibrium.
- Wash the beads sequentially with washing buffers 1, 2, and 3 to remove nonspecifically bound proteins.
- Elute the SUMO-GFP11 protein with elution buffer.
- Dialyze the eluted solution with binding buffer and concentrate using an Amicon 10K ultrafiltration tube.
- Confirm the purity of SUMO-GFP11 protein by SDS-PAGE.

1. **Determination of SUMO-GFP11 concentration.**

- Measure the protein concentration using a NanoDrop spectrophotometer at 280 nm.

**2.3 Purification of GFP1-9 protein:** The purification of GFP1-9 was performed according to a method reported by Jiang et al., 2015^2^.

**Reagent preparation:**

Binding buffer: 300 mM NaCl, 10% glycerol, 50 mM Tris-HCl (pH 8.0).

Washing buffer 1: 300 mM NaCl, 10% glycerol, 50 mM Tris-HCl (pH 8.0) with 20 mM imidazole

Washing buffer 2: 300 mM NaCl, 10% glycerol, 50 mM Tris-HCl (pH 8.0) with 50 mM imidazole

Washing buffer 3: 300 mM NaCl, 10% glycerol, 50 mM Tris-HCl (pH 8.0) with 80 mM imidazole

Elution buffer: 300 mM NaCl, 10% glycerol, 50 mM Tris-HCl (pH 8.0) with 500 mM imidazole.

Loading buffer: 20 mM HEPES (pH 7.5) with 500 mM NaCl.

Running buffer: 50 mM Tris-HCl (pH 8.0) with 300 mM NaCl.

Denaturing buffer: 50 mM Tris-HCl buffer (pH 8.0) with 300 mM NaCl and 3 M guanidine HCl.

Refolding buffer: 50 mM Tris-HCl ( pH 8.0) with 10% glycerol and 100 mM NaCl.

**Purification steps:**

1. **Bacteria inoculation.**

- Inoculate a single colony of *E. coli* BL21(DE3) carrying the pET28a-GFP1-9-3C-10-3C-11 plasmid into 10 mL of LB medium supplemented with 50 μg/mL kanamycin.
- Incubate the culture overnight on a shaking table at 37 °C and 220 rpm.

1. **Bacteria culture.**

- Use the overnight culture to inoculate 800 mL of fresh LB medium (inoculation volume of 1%) containing 50 μg/mL kanamycin.
- Incubate the culture at 37 °C with shaking at 220 rpm.
- Add IPTG to a final concentration of 0.5 mM when the culture reaches an OD600 of 0.6 - 0.8.
- Allow the *E. coli* cells to express the recombinant GFP1-9-3C-10-3C-11 at 19 °C for 20 hours.

1. **Acquisition of GFP1-9 protein** **crude extracts.**

- Centrifuge the cells at 4500 rpm for 15 minutes to collect them.
- Resuspend the pellet in 15 mL of binding buffer.
- Ultrasonicate the cell suspension on ice for 40 minutes (1 s ON and 3 s OFF).
- Centrifuge the lysate at 12000 rpm at 4 °C for 60 minutes.

1. **Full-length GFP purification.**

- Incubate the supernatant with Ni-NTA beads for 1 hour at 4°C.
- Wash the beads sequentially with washing buffers 1, 2, and 3 to remove undesired proteins.
- Elute the GFP1-9-3C-10-3C-11 protein (full-length GFP) with elution buffer.
- Concentrate and wash the eluted solution with binding buffer in a 30K ultrafiltration tube to remove excess imidazole.

1. **Cleavage of full-length GFP.**

- Treat the concentrated protein with 3C protease (with a GST tag) at a concentration of 1 U/ml overnight at 4 °C.
- Mix the protein solution with GST beads (GE Healthcare) at 4 °C for 1 hour to remove 3C protease, followed by washing with loading buffer.
- Collect the proteins that flow through the column and concentrate to a volume of 1 mL.
- Load the protein solution onto a Superdex 75 size exclusion column pre-equilibrated with running buffer to remove extra 3C protease.

1. **GFP1-9 purification.**

- Collect the fraction tubes with GFP1-9 protein.
- Denature GFP1-9 protein in 3 M guanidine HCl at 4 °C for 2 hours.
- Separate GFP1-9 from GFP10-11 on a Superdex75 size exclusion column in denaturing buffer.
- Refold GFP1-9 by dialysis against refolding buffer at 4 °C overnight.
- Confirm the purity of GFP1-9 protein by SDS-PAGE.

1. **Determination of GFP1-9 concentration.**

- Measure the concentration using a NanoDrop spectrophotometer at 280 nm.

**2.4 Purification of ubiquitin-GFP10-11 protein**

**Reagent preparation:**

Binding buffer: 300 mM NaCl, 10% glycerol, 50 mM Tris-HCl buffer (pH 8.0).

Washing buffer 1: 300 mM NaCl, 10% glycerol, 50 mM Tris-HCl buffer (pH 8.0) with 20 mM imidazole.

Washing buffer 2: 300 mM NaCl, 10% glycerol, 50 mM Tris-HCl buffer (pH 8.0) with 50 mM imidazole.

Washing buffer 3: 300 mM NaCl, 10% glycerol, 50 mM Tris-HCl buffer (pH 8.0) with 80 mM imidazole.

Elution buffer: 300 mM NaCl, 10% glycerol, 50 mM Tris-HCl buffer (pH 8.0) with 500 mM imidazole.

**Purification steps:**

1. **Bacteria inoculation.**

- Inoculate a single colony of *E. coli* BL21(DE3) carrying the pET24a-ubiquitin-GFP10-11 plasmid into 10 mL of LB medium supplemented with 50 μg/mL kanamycin.
- Incubate the culture overnight on a shaking table at 37 °C and 220 rpm.

1. **Bacteria culture.**

- Use the overnight culture to inoculate 800 mL of fresh LB medium (inoculation volume of 1%) containing 50 μg/mL kanamycin.
- Incubate the culture at 37 °C and 220 rpm.
- Add IPTG to a final concentration of 1.0 mM when the culture reaches an OD600 of 0.6 - 0.8.
- Allow the *E. coli* cells to express ubiquitin-GFP10-11 at 19 °C for 20 hours.

1. **Acquisition of ubiquitin-GFP10-11 protein** **crude extracts.**

- Centrifuge the cells at 4500 rpm for 15 minutes to collect them.
- Resuspend the pellet in 15 mL of binding buffer.
- Ultrasonicate the cell suspension on ice for 40 minutes (1 s ON and 3 s OFF).
- Centrifuge the lysate at 12000 rpm at 4 °C for 60 minutes.

1. **Ubiquitin-GFP10-11 purification** .

- Incubate the supernatant with Ni-NTA beads for 1 hour at 4°C.
- Wash the beads sequentially with washing buffers 1, 2, and 3 to remove nonspecifically bound proteins.
- Elute the ubiquitin-GFP10-11 protein with elution buffer.
- Concentrate and wash eluted solution with binding buffer in a 10K ultrafiltration tube to remove excess imidazole.
- Load 1 mL of the concentrated ubiquitin-GFP10-11 onto a Superdex 75 size exclusion column pre-equilibrated with binding buffer.
- Collect the ubiquitin-GFP10-11 protein fractions.
- Confirm the purity of ubiquitin-GFP10-11 protein by SDS-PAGE.

1. **Determination of Ubiquitin-GFP10-11 concentration.**

- Measure the protein concentration using a NanoDrop spectrophotometer at 280 nm.

**Supplementary Tables**

Table S1 Comparison of existing methods with split-GFP assembly method for visualizing displayed proteins

|  | Characteristics | Passenger protein antibody | Tag antibody | GFP nanobody | Display of fluorescent protein | GFP1-10/GFP11 assembly | GFP1-9/GFP10-11 assembly |
| --- | --- | --- | --- | --- | --- | --- | --- |
| Basic information | Cost | High | High | Low | Low | Low | Low |
|  | Tag size | - | 6-10 aa | 111 aa | 238 a.a. | 16 a.a. | 33 a.a. |
|  | Fluorescence background | Low | Low | Low | High | Low | High |
| Supplementary protein preparation | Time required | weeks to months | weeks to months | 1 day | - | 7 hours | 5 days |
|  | Purification steps | Complicated | Complicated | Simple | Simple | Simple | Complicated |
| Incubation parameters | Incubation time | ~2h | ~2h | 0.5-2h | - | 12 h | 3 h |
|  | Incubation condition | 4 ℃, dark | 4 ℃, dark | - | - | Room temperature | Room temperature |
| Quantification |  | No | No | No | No | Yes | Yes |
| Reference |  | 3-4 | 5-11 | 12 | 9, 13-14 | This study | This study |

Table S2 The nucleotide sequences encoding the split-GFP fragments

| Name | Sequence |
| --- | --- |
| GFP1-10 | ATGCATCATCATCATCATCACAGCAGCGGCGCTAGCATGTCCAAAGGAGAAGAACTGTTTACCGGTGTTGTGCCAATTTTGGTTGAACTCGATGGTGATGTCAACGGACATAAGTTCTCAGTGAGAGGCGAAGGAGAAGGTGACGCCACCATTGGAAAATTGACTCTTAAATTCATCTGTACTACTGGTAAACTTCCTGTACCATGGCCGACTCTCGTAACAACGCTTACGTACGGAGTTCAGTGCTTTTCGAGATACCCAGACCATATGAAAAGACATGACTTTTTTAAGTCGGCTATGCCTGAAGGTTACGTGCAAGAAAGAACAATTTCGTTCAAAGATGATGGAAAATATAAAACTAGAGCAGTTGTTAAATTTGAAGGAGATACTTTGGTTAACCGCATTGAACTGAAAGGAACAGATTTTAAAGAAGATGGTAATATTCTTGGACACAAACTCGAATACAATTTTAATAGTCATAACGTATACATCACTGCTGATAAGCAAAAGAACGGAATTAAAGCGAATTTCACAGTACGCCATAATGTAGAAGATGGCAGTGTTCAACTTGCCGACCATTACCAACAAAACACCCCTATTGGAGACGGTCCGGTACTTCTTCCTGATAATCACTACCTCTCAACACAAACAGTCCTGAGCAAAGATCCAAATGAAAAATAG |
| GFP1-9-3C-10-3C-11 | ATGTCCAAAGGAGAAGAACTGTTTACCGGTGTTGTGCCAATTTTGGTTGAACTCGATGGTGATGTCAACGGACATAAGTTCTCAGTGAGAGGCGAAGGAGAAGGTGACGCCACCATTGGAAAATTGACTCTTAAATTCATCTcaACTACTGGTAAACTTCCTGTACCATGGCCGACTCTCGTAACAACGCTTACGTACGGAGTTCAGgcCTTTTCGAGATACCCAGACCATATGAAAAGACATGACTTTTTTAAGTCGGCTATGCCTGAAGGTTACGTGCAAGAAAGAACAATTTCGTTCAAAGATGATGGAAAATATAAAACTAGAGCAGTTGTTAAATTTGAAGGAGATACTTTGGTTAACCGCATTGAACTGAAAGGAACAGATTTTAAAGAAGATGGTAATATTCTTGGACACAAACTCGAATACAATTTTAATAGTCATAACGTATACATCACTGCTGATAAGCAAAAGAACGGAATTAAAGCGAATTTCACAGTACGCCATAATGTAGAAGATGGCAGTGTTCAACTTGCCGACCATTACCAACAAAACACCCCTATTGGAGACGGTCTGGAAGTTCTGTTCCAGGGGCCCTCAGGTTCAGGTTCACCGGTACTTCTTCCTGATAATCACTACCTCTCAACACAAACAGTCCTGAGCAAAGATCCAAATGAAGCACTGGAAGTTCTGTTCCAGGGGCCCTCAGGTTCAGGTTCACGCGATCACATGGTCCTGCACGAGTACGTGAACGCCGCCGGGATCACTCATGGTATGGATGAACTGTATAAAGGTAGCGGTGGTACCTAG |
| SUMO-GFP11 | ATGTCGGACTCAGAAGTCAATCAAGAAGCTAAGCCAGAGGTCAAGCCAGAAGTCAAGCCTGAGACTCACATCAATTTAAAGGTGTCCGATGGATCTTCAGAGATCTTCTTCAAGATCAAAAAGACCACTCCTTTAAGAAGGCTGATGGAAGCGTTCGCTAAAAGACAGGGTAAGGAAATGGACTCCTTAAGATTCTTGTACGACGGTATTAGAATTCAAGCTGATCAGACCCCTGAAGATTTGGACATGGAGGATAACGATATTATTGAGGCTCACAGAGAACAGATTGGTGGAGATGGAGGGTCTGGTGGCGGATCACGCGATCACATGGTCCTGCACGAGTACGTGAACGCCGCCGGGATCACTTAG |
| Ubiquitin-GFP10-11 | ATGCAGATCTTCGTGAAGACCCTGGGCGGCGGCGGCGGCAACCATTATCTGAGCACCCAGACCGTGCTGAGCAAAGATCCGAACGAAAAACGCGATCACATGGTCCTGCACGAGTACGTGAACGCCGCCGGGATCACTGGCGGCGGCGGCACTGGTAAGACCATCACTCTCGAAGTGGAGCCGAGTGACACCATTGAGAATGTCAAGGCAAAGATCCAAGACAAGGAAGGCATCCCTCCTGACCAGCAGAGGTTGATCTTTGCTGGGAAACAGCTGGAAGATGGACGCACCCTGTCTGACTACAACATCCAGAAAGAGTCCACCCTGCACCTGGTGCTCCGTCTTAGAGGTGGGCTCGAG |
| Lpp-OmpA | ATGAAAGCTACTAAACTGGTACTGGGCGCGGTAATCCTGGGTTCTACTCTGCTGGCAGGTTGCTCCAGCAACGCTAAAATCGATCAGAACAATGGCCCGACCCATGAAAACCAACTGGGCGCTGGTGCTTTTGGTGGTTACCAGGTTAACCCGTATGTTGGCTTTGAAATGGGTTACGACTGGTTAGGTCGTATGCCGTACAAAGGCAGCGTTGAAAACGGTGCATACAAAGCTCAGGGCGTTCAACTGACCGCTAAACTGGGTTACCCAATCACTGACGACCTGGACATCTACACTCGTCTGGGTGGCATGGTATGGCGTGCAGACACTAAATCCAACGTTTATGGTAAAAACCACGACACCGGCGTTTCTCCGGTCTTCGCTGGCGGTGTTGAGTACGCGATCACTCCTGAAATCGCTACCCGT |
| OmpC | ATGAAAGTTAAAGTACTGTCCCTCCTGGTCCCAGCTCTGCTGGTAGCAGGCGCAGCAAACGCTGCTGAAGTTTACAACAAAGACGGCAACAAATTAGATCTGTACGGTAAAGTAGACGGCCTGCACTATTTCTCTGACAACAAAGATGTAGATGGCGACCAGACCTACATGCGTCTTGGCTTCAAAGGTGAAACTCAGGTTACTGACCAGCTGACCGGTTACGGCCAGTGGGAATATCAGATCCAGGGCAACAGCGCTGAAAACGAAAACAACTCCTGGACCCGTGTGGCATTCGCAGGTCTGAAATTCCAGGATGTGGGTTCTTTCGACTACGGTCGTAACTACGGCGTTGTTTATGACGTAACTTCCTGGACCGACGTACTGCCAGAATTCGGTGGTGACACCTACGGTTCTGACAACTTCATGCAGCAGCGTGGTAACGGCTTCGCGACCTACCGTAACACTGACTTCTTCGGTCTGGTTGACGGCCTGAACTTTGCTGTTCAGTACCAGGGTAAAAACGGCAACCCATCTGGTGAAGGCTTTACTAGTGGCGTAACTAACAACGGTCGTGACGCACTGCGTCAAAACGGCGACGGCGTCGGCGGTTCTATCACTTATGATTACGAAGGTTTCGGTATCGGTGGTGCGATCTCCAGCTCCAAACGTACTGATGCTCAGAACACCGCTGCTTACATCGGTAACGGCGACCGTGCTGAAACCTACACTGGTGGTCTGAAATACGACGCTAACAACATCTACCTGGCTGCTCAGTACACCCAGACCTACAACGCAACTCGCGTAGGTTCCCTGGGTTGGGCGAACAAAGCACAGAACTTCGAAGCTGTTGCTCAGTACCAGTTC |
| InaZ | ATGAACCTGGATAAAGCCCTGGTGCTGCGCACCTGTGCAAACAACATGGCAGATCATTGTGGTCTGATTTGGCCGGCCAGCGGCACCGTTGAAAGCCGTTATTGGCAGAGCACCCGTCGTCATGAAAATGGTCTGGTTGGTCTGCTGTGGGGTGCAGGTACAAGCGCATTTCTGAGTGTTCATGCGGATGCTCGTTGGATTGTGTGTGAAGTTGCAGTTGCGGATATTATTTCTCTGGAAGAACCTGGTATGGTTAAATTTCCTCGTGCGGAAGTTGTTCATGTTGGTGATCGTATTAGCGCAAGCCATTTTATTTCTGCGCGTCAGGCAGATCCGGCAAGCACCAGCACCTCAACCCTGACCCCGATGCCGACCGCCATTCCTACCCCGATGCCTGCAGTTGCAAGCGTTACCCTGCCAGTTGCAGAACAGGCCCGTCATGAAGTTTTTGATGTAGCAAGCGTTTCAGCCGCAGCAGCACCTGTTAATACCCTGCCGGTTACCACCCCGCAGAATGTTCAGACAGCAACCTATGGTAGTCAT |

Note: The 3C protease recognized sequence is marked in red, and the linker sequence is marked in orange.

Table S3 Primers used in this study

| Primer | Sequence |
| --- | --- |
| pZL1 | AAGTATAAGAAGGAGATATACATATGATGAAAGCTACTAAACTGGTAC |
| pZL2 | CGCTACCCGTATGTCGGACTCAGAAGTCAATC |
| pZL3 | AGTCCGACATACGGGTAGCGATTTCAGGAG |
| pZL4 | GCAGCGGTTTCTTTACCAGACTCGAGCTAAGTGATCCCGGCGGCGTTC |
| pZL5 | GTATAAGAAGGAGATATACATATGATGAAAGTTAAAGTACTGTCC |
| pZL6 | AGTCCGACATGAACTGGTACTGAGCAACAG |
| pZL7 | GTACCAGTTCATGTCGGACTCAGAAGTCAATC |
| pZL8 | AAGTATAAGAAGGAGATATACATATGATGAACCTGGATAAAGCCCT |
| pZL9 | AGTCCGACATATGACTACCATAGGTTGCTG |
| pZL10 | TGGTAGTCATATGTCGGACTCAGAAGTCAAT |
| pZL11 | CGCTACCCGTATGACACTTGAAAAATTTGTG |
| pZL12 | ACCCTCCATCTTTATGGGGATCAGTTATATC |
| pZL13 | TCCCCATAAAGATGGAGGGTCTGGTGGCGG |
| pZL14 | CAAGTGTCATACGGGTAGCGATTTCAGGAGTG |
| pZL15 | CAAGTGTCATGAACTGGTACTGAGCAACAG |
| pZL16 | GTACCAGTTCATGACACTTGAAAAATTTGTG |
| pZL17 | TGGTAGTCATATGACACTTGAAAAATTTGTG |
| pZL18 | CAAGTGTCATATGACTACCATAGGTTGCTG |
| pZL19 | CGCTACCCGTATGCAGATCTTCGTGAAGAC |
| pZL20 | CAGACTCGAGCTACTCGAGCCCACCTCTAA |
| pZL21 | GCTCGAGTAGCTCGAGTCTGGTAAAGAAAC |
| pZL22 | AGATCTGCATACGGGTAGCGATTTCAGGAG |
| pZL23 | GTACCAGTTCATGCAGATCTTCGTGAAGAC |
| pZL24 | AGATCTGCATGAACTGGTACTGAGCAACAG |
| pZL25 | TGGTAGTCATATGCAGATCTTCGTGAAGAC |
| pZL26 | AGATCTGCATATGACTACCATAGGTTGCTG |
| pZL27 | CGCTACCCGTATGACACTTGAAAAATTTGTG |
| pZL28 | GATAATGGTTTGATCCGCCACCAGACCCTCCATCTTTATGGGGATCAGTTATATCC |
| pZL29 | GTACCAGTTCATGACACTTGAAAAATTTGTG |
| pZL30 | TGGTAGTCATATGACACTTGAAAAATTTGTG |
| pZL31 | TCCCCATAAAGATGGAGGGTCTGGTGGCGGATCAAACCATTATCTGAGCACCCAG |
| pZL32 | CAAGTGTCATACGGGTAGCGATTTCAGGAGTG |
| pZL33 | CAAGTGTCATGAACTGGTACTGAGCAACAG |
| pZL34 | CAAGTGTCATATGACTACCATAGGTTGCTG |

**Supplementary Figures**


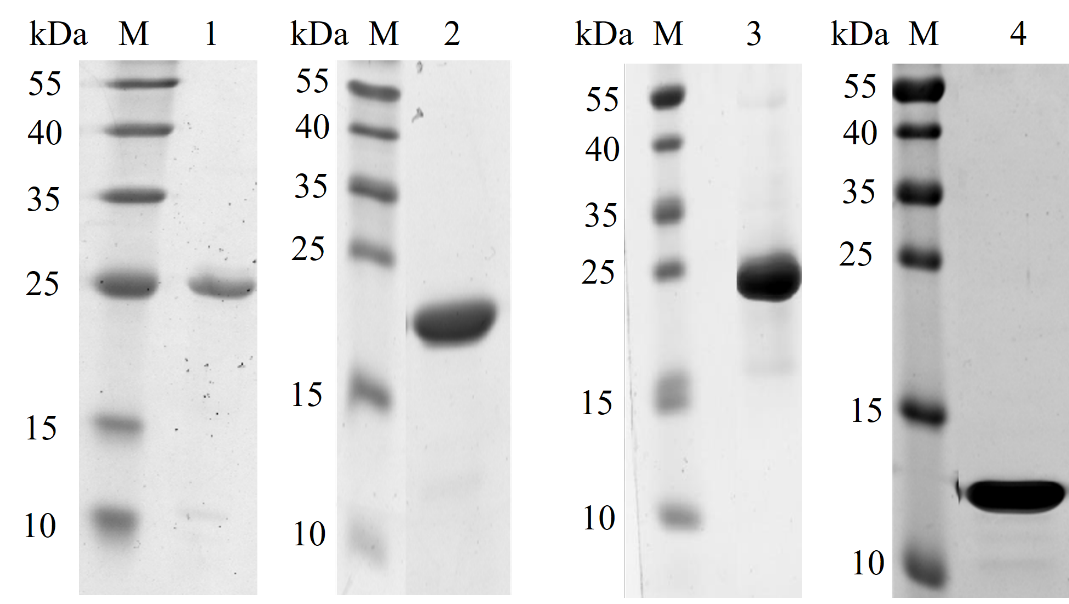


Fig. S1 SDS-PAGE analysis of purified GFP1-10 (lane 1, 25.4 kDa), SUMO-GFP11 (lane 2, 16.1 kDa), GFP1-9 (lane 3, 22.7 kDa), and Ubiquitin-GFP10-11 (lane 4, 14 kDa).


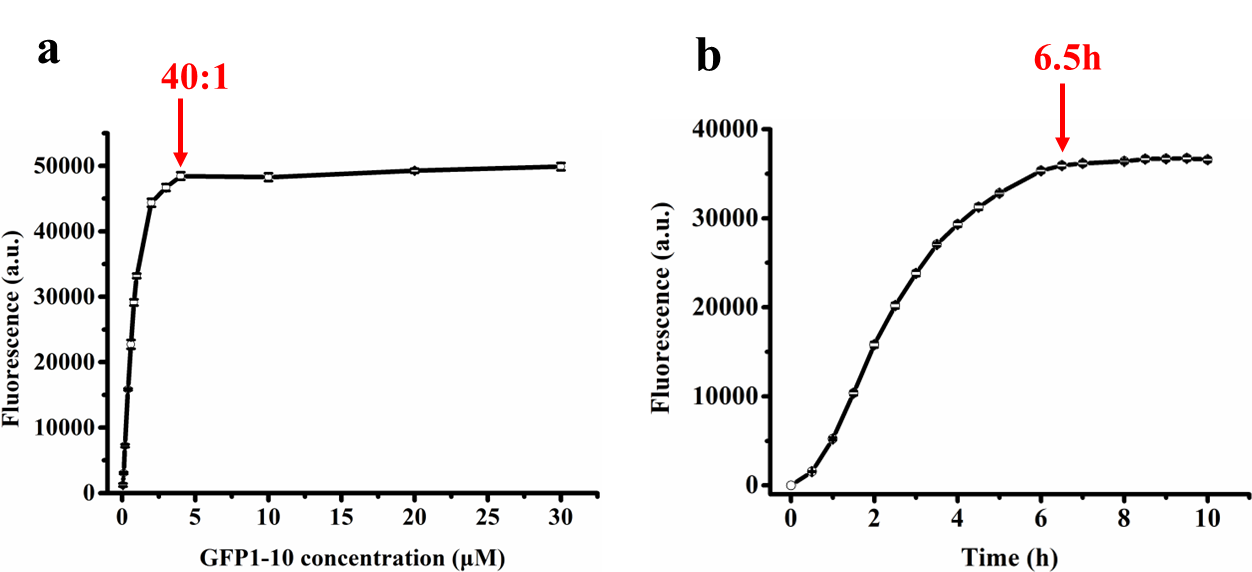


Fig. S2. Optimization of GFP1-10/SUMO-GFP11 incubation conditions. (a) The fluorescence intensity versus the ratio of GFP1-10 to SUMO-GFP11 after 12 hours’ incubation; (b) Time-profile of fluorescence intensity during the assembly of GFP1-10 and SUMO-GFP11.


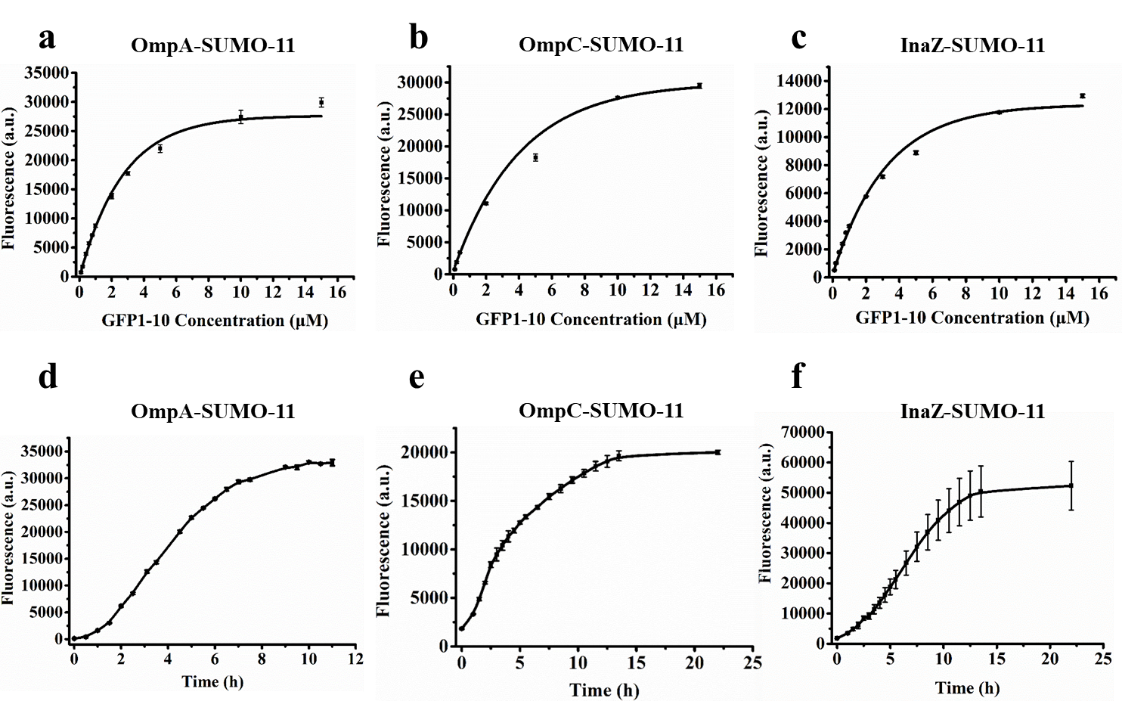


Fig. S3. Optimization of the incubation conditions of GFP1-10 with cells displaying SUMO-GFP11. (a-c) The fluorescence intensity versus the ratio of GFP1-10 to surface displaying cells; (d-f) Time-profile of fluorescence intensity during the assembly of GFP1-10 and surface displaying cells.


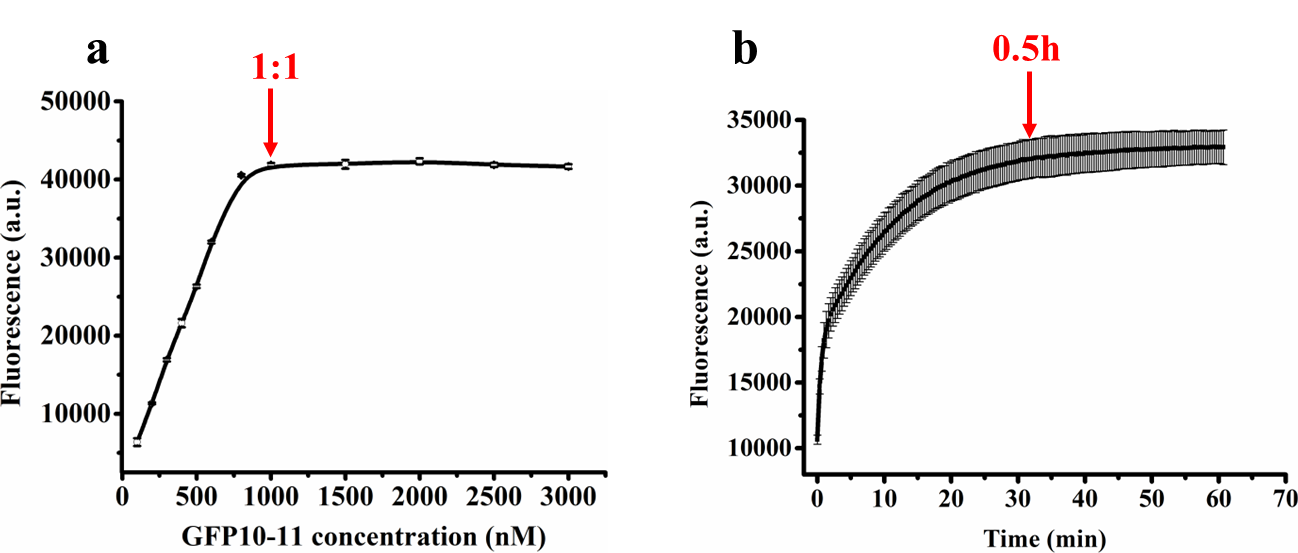


Fig. S4. Optimization of GFP1-9/Ubiquitin-GFP10-11 incubation conditions. (a) The fluorescence intensity versus the ratio of GFP1-9 to Ubiquitin-GFP10-11 after 3 hours’ incubation; (b) Time-profile of the fluorescence intensity during the assembly of GFP1-9 and Ubiquitin-GFP10-11.


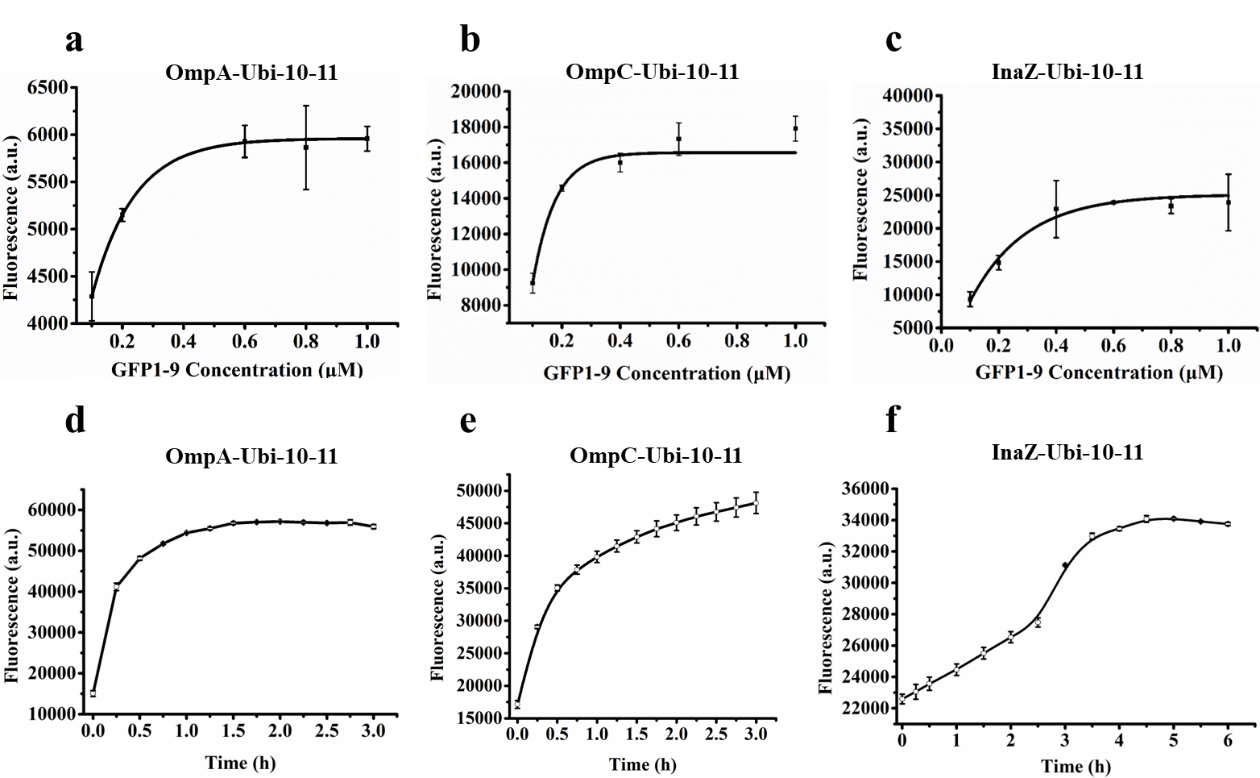


Fig. S5. Optimization of the incubation conditions of GFP1-9 with cells displaying ubiquitin-GFP10-11. (a-c) the fluorescence intensity versus the ratio of GFP1-9 to surface displaying cells. (d-f) time-profile of the fluorescence intensity during assembly of GFP1-9 with surface displaying cells.

Fig. S6. The background fluorescence of OmpA-Ubi-GFP10-11, OmpC-Ubi-GFP10-11, and InaZ-Ubi-GFP10-11 after incubation with GFP1-9 at different concentration at time 0.

**References**

1. Cabantous S, Waldo GS. In vivo and in vitro protein solubility assays using split GFP. Nat Methods. 2006; 3: 845-54.

2. Jiang WX, Dong X, Jiang J, Yang YH, Yang J, Lu YB,et al. Specific cell surface labeling of GPCRs using split GFP. Sci Rep. 2016; 6: 20568-76.

3. Sungkeeree P, Whangsuk W, Dubbs J, Mongkolsuk S, Loprasert S: Biodegradation of endocrine disrupting dibutyl phthalate by a bacterial consortium expressing *Sphingobium* sp. SM42 esterase. Process Biochem. 2016; 51: 1040-45.

4. Zhang Z, Liu J, Fan J, Wang Z, Li L.Detection of catechol using an electrochemical biosensor based on engineered *Escherichia coli* cells that surface-display laccase. Analytica Chimica Acta 2018; 1009: 65-72.

5. Chen Z, Duan R, Xiao Y, Wei Y, Zhang H, Sun X, et al. Biodegradation of highly crystallized poly(ethylene terephthalate) through cell surface codisplay of bacterial PETase and hydrophobin. Nat Commun. 2022; 13: 7138-54.

6. Liang B, Liu Y, Zhao Y, Xia T, Chen R, Yang J. Development of bacterial biosensor for sensitive and selective detection of acetaldehyde. Biosens Bioelectron. 2021; 193: 113566-75.

7. Nicchi S, Giuliani M, Giusti F, Pancotto L, Maione D, Delany I, et al. Decorating the surface of *Escherichia coli* with bacterial lipoproteins: a comparative analysis of different display systems. Microb Cell Fact. 2021; 20: 33-46.

8. Cherf GM, Cochran JR. Applications of yeast surface display for protein engineering. Methods Mol Biol. 2015; 1319: 155-75.

9. Zahradnik J, Dey D, Marciano S, Kolarova L, Charendoff CI, Subtil A, et al. A protein-engineered, enhanced yeast display platform for rapid evolution of challenging targets. ACS Synth Biol. 2021; 10: 3445-60.

10. Uchanski T, Zogg T, Yin J, Yuan D, Wohlkonig A, Fischer B, et al. An improved yeast surface display platform for the screening of nanobody immune libraries. Sci Rep. 2019; 9: 382-93.

11. Wang X, Qiao O, Han L, Li N, Gong Y. A novel rabbit anti-myoglobin monoclonal antibody’s potential application in rhabdomyolysis associated acute kidney injury. Int J Mol Sci. 2023; 24: 7822-45.

12. Wendel S, Fischer EC, Martinez V, Seppala S, Norholm MH. A nanobody: GFP bacterial platform that enables functional enzyme display and easy quantification of display capacity. Microb Cell Fact. 2016; 15: 71-83.

13. Feilmeier BJ, Iseminger G, Schroeder D, Webber H, Phillips GJ. Green fluorescent protein functions as a reporter for protein localization in *Escherichia coli*. J Bacteriol. 2000; 182: 4068-76.

14. Peter O, Srikant-Iyer S, Lange S, Schmitt J, Schmid RD. Fluorophor-linked immunosorbent assay: a time- and cost-saving method for the characterization of antibody fragments using a fusion protein of a single-chain antibody fragment and enhanced green fluorescent protein. Anal Biochem. 2002; 309: 27-34.
